# Structural and tribometric characterization of biomimetically inspired synthetic "insect adhesives"

**DOI:** 10.3762/bjnano.8.6

**Published:** 2017-01-06

**Authors:** Matthias W Speidel, Malte Kleemeier, Andreas Hartwig, Klaus Rischka, Angelika Ellermann, Rolf Daniels, Oliver Betz

**Affiliations:** 1 Institut für Evolution und Ökologie, Universität Tübingen, Auf der Morgenstelle 28, D-72076 Tübingen, Germany; 2 Fraunhofer-Institut für Fertigungstechnik und Angewandte Materialforschung, Wiener Str. 12, D-28359 Bremen, Germany; 3 Universität Bremen, Fachbereich 2 Biologie/Chemie, Leobener Str., 28359 Bremen, Germany; 4 Pharmazeutisches Institut, Universität Tübingen, Pharmazeutische Technologie und Biopharmazie, Auf der Morgenstelle 8, D-72076 Tübingen, Germany

**Keywords:** adhesion, bionics, emulsion, friction, insects

## Abstract

**Background:** Based on previous chemical analyses of insect tarsal adhesives, we prepared 12 heterogeneous synthetic emulsions mimicking the polar/non-polar principle, analysed their microscopical structure and tested their adhesive, frictional, and rheological properties.

**Results:** The prepared emulsions varied in their consistency from solid rubber-like, over soft elastic, to fluid (watery or oily). With droplet sizes >100 nm, all the emulsions belonged to the common type of macroemulsions. The emulsions of the first generation generally showed broader droplet-size ranges compared with the second generation, especially when less defined components such as petrolatum or waxes were present in the lipophilic fraction of the first generation of emulsions. Some of the prepared emulsions showed a yield point and were Bingham fluids. Tribometric adhesion was tested via probe tack tests. Compared with the "second generation" (containing less viscous components), the "first generation" emulsions were much more adhesive (31–93 mN), a finding attributable to their highly viscous components, i.e., wax, petrolatum, gelatin and poly(vinyl alcohol). In the second generation emulsions, we attained much lower adhesivenesses, ranging between 1–18 mN. The adhesive performance was drastically reduced in the emulsions that contained albumin as the protein component or that lacked protein. Tribometric shear tests were performed at moderate normal loads. Our measured friction forces (4–93 mN in the first and 0.1–5.8 mN in the second generation emulsions) were comparatively low. Differences in shear performance were related to the chemical composition and emulsion structure.

**Conclusion:** By varying their chemical composition, synthetic heterogeneous adhesive emulsions can be adjusted to have diverse consistencies and are able to mimic certain rheological and tribological properties of natural tarsal insect adhesives.

## Introduction

During evolution, insects have developed the ability to move vertically and upside-down on various kinds of surface, a feat that has facilitated their successful exploration of a huge diversity of habitats. In this context, insects have evolved two distinctly different mechanisms to attach themselves to a variety of substrates, i.e., hairy surfaces and smooth flexible pads [1]. Usually, both types of adhesive devices involve supplementary adhesive fluids produced by glandular systems underlying the adhesive cuticular structures [2–4]. One major function of these liquid adhesives is to wet and maximize the contact area with the substrate by filling its surface irregularities [5–6]. In addition, viscous and capillary forces are conveyed by the adhesive secretion [7–12]. Recently, the suggestion has been made, that during friction regimes, insect adhesives induce rate-dependent viscosity changes caused by non-Newtonian shear strains [5,13–14].

Chemical analyses of adhesive insect secretions employed during locomotion have revealed that they form heterogeneous (emulsion-like) mixtures of aliphatic lipids, carbohydrates and proteins [4,15–19]. Adhesive secretions may form both oil-in-water (o/w) [20–22] or water-in-oil (w/o) emulsions [14,23]. Possible functional advantages lie in (i) their increased flexibility towards substrates of different surface energy and polarity, (ii) their possible non-Newtonian viscosity shifts implying adjustable viscosities [24–25] and (iii) the formation of lipoid shields that prevent the aqueous fraction of an adhesive from desiccation and its sticking to the walls of the outlet ductule [4,20,26]. Moreover, within the lipoid fraction itself, both the specific constitution and the mixing ratio of the various hydrocarbon molecules might also largely influence their adhesive performance possibly via viscosity and surface tension effects, molecular re-orientations and the intermolecular attraction of the hydrocarbon chains in the thin liquid films [27]. In hydrocarbon molecules, viscosity is positively correlated with chain length, whereas the degree of unsaturation and the number of double bonds and methyl branches have the opposite effect. The position of the double bonds and the methyl branches modify this behaviour [27]. Indeed, in the potato beetle *Leptinotarsa decemlineata*, the supplementation of unsaturated components (e.g., *cis*-alkenes) to the adhesive tarsal secretions results in a significant reduction of friction forces [28].

Recent chemical analyses of the tarsal adhesives of the locust *Schistocerca gregarina* [15] and the Madagascan hissing cockroach *Gromphadorrhina portentosa* [16–17] have confirmed that the lipoid phase of their adhesives consists of *n*-alkanes (in the range of C_23_–C_49_ in *S. gregaria* and C_27_–C_34_ in *G. portentosa*), internally branched monomethyl-, dimethyl- and trimethyl- (the latter substance in *S. gregaria* only) alkanes and long-chain fatty acids and aldehydes (in *S. gregaria* only). In the tarsal adhesives of the hairy adhesive systems of the frog beetle *Sagra femorata* and carrion beetles of the genus *Nicrophorus,* Gerhardt et al. [17] have established a hydrocarbon spectrum in the C-range between C_18_ and C_39_ including *n*-alkanes, mono-, di-, tri- and tetramethyl branched alkanes, alkenes, alkadienes and one aldehyde (the latter substance in *S. femortata* only).

The established long-chain *n*-alkanes suggest a semi-solid (grease-like) consistency of the adhesive emulsion with increased viscosity, in accordance with the properties of a Bingham fluid. Such a property would consolidate several functional principles and properties that are essential for effective locomotion: (1) improving slip resistance, (2) facilitating subsequent tarsal release from the substrate, (3) reducing the loss of tarsal fluid on the substrate, (4) keeping the adhesive compliable for perfect adaption to the surface micro-roughness and (5) protecting of the tarsal adhesive pads from contamination and abrasive damage [15]. In addition, our recently performed analyses [15,18] have confirmed the presence of polysaccharides, peptides and (glycosylated) proteins in the adhesive secretion of the desert locust *Schistocerca gregaria* and the Madagascan hissing cockroach *Gromphadorhina portentosa* and have thus confirmed previous assumptions of Vötsch et al. [21].

Although the analysis of the structure and the function of the emulsion-like adhesives of insects is still in its infancy, these adhesives combine interesting properties relevant for possible commercial applications and the development of biomimetically inspired lipid-based adhesives. We have used the available chemical data [4,15–18,21] concerning the heterogeneous composition of insect tarsal secretions to prepare synthetic adhesives based upon the biological "polar/non-polar" principle. We have investigated the way that both the adhesive and the frictional performance of the adhesive are influenced by the co-occurrence of highly polar and highly non-polar components in combination with amphiphilic substances, whereby our preparations have covered both liquid and semi-solid (grease-like) alternatives. Such heterogeneously assembled emulsions have the potential of being optimized with respect to certain required properties.

For the preparation of synthetic "insect adhesives", we abstracted the chemical components revealed in the chemical analyses of the insect examples mentioned above by replacing their components by low-priced and simply producible natural or synthetic compounds of comparable structure and properties. We developed two consecutive generations (designated as "first" and "second" generation) of synthetic emulsions mimicking the lipid-based "polar/non-polar" insect example. In the first generation (containing highly viscous components), the non-polar phase was represented by microcrystalline wax or petrolatum (trademark Vaseline) consisting of hydrocarbons of various lengths (C_30_–C_70_) and branching positions. The aqueous polar phase was enriched by the water-soluble protein/peptide mixture gelatin or poly(vinyl alcohol), which is considered as a carbohydrate equivalent because of its numerous hydroxy groups. In our preparations, the gelatin was plasticized by the addition of glycerine.

Our second generation of synthetic "insect adhesives" (containing less viscous components) consisted of the *n*-alkane octacosane (C_28_) and the hexamethyl-alkane squalane (C_30_) representing hydrocarbons of defined structure and length within the range of C_23_–C_49_ as established in the biological role models. Albumin and gelatin were substitutes for proteinogenic amino acids, with the surfactant Span 80 (Sorbitane monooleate**)** being used as a combined replacement for fatty acids and carbohydrates. The general composition of the emulsions and assignment to the used abbreviations is summarized in Table 1. Although emulsions are very common in technical applications and have extensively been examined, it is still not trivial to properly prepare and characterize them. The reason is that emulsions are not in a thermodynamic equilibrium, but rather represent a “frozen state”, which depends not only on their composition, but also strongly on their way of preparation [29–31]. This is especially the case for adjusting their non-Newtonian rheological behaviour and their droplet distribution. The purpose of our contribution is not to present completely new kinds of emulsion, but to use emulsions in a biomimetic context. Due to the small amounts of attainable natural tarsal secretions, it is hardly possible to determine their droplet sizes and other emulsion parameters. Therefore, the artificial emulsions prepared and used in the present contribution are used as rough models to indirectly deduce how the biological adhesives are probably structured and how they perform. This is the reason why we follow a typical process sequence of biomimetic research [32], i.e., we intend to mimick tribological properties of tarsal insect adhesives by preparing a second generation of technical emulsions on the basis of insights gained from a first (more imperfect) generation. Since natural tarsal adhesive emulsions of insects are hard to isolate and thus not well accessible to experimental approaches, our "biomimetic approach" will help to (i) understand possible structural and functional principles that make up such adhesives and (ii) develop technical protocols how to test and mimick them. On a long term perspective, such approaches will help to technically utilize insect tarsal adhesives principles.

**Table 1:** List of the prepared synthetic emulsions and their main composition.

generation	name of emulsion	hydrocarbon	emulsifier^a^	protein	carbohydrate equivalent	amphiphilic compound (fatty acid + carbohydrate)^b^	consistency at room temperature (22 °C)	emulsion character^c^

1	VG50	petrolatum	SDS	gelatin solution	—	—	solid	o/w
1	VP50	petrolatum	SDS	—	poly(vinyl alcohol) solution	—	soft	o/w
1	WG20	microcrystalline wax	SDS	gelatin solution	—	—	solid	o/w
1	WP20	microcrystalline wax	SDS	—	poly(vinyl alcohol) solution	—	solid	o/w
2	SA2	squalane	SDS	albumin	—	Span 80	liquid	o/w
2	SG2	squalane	SDS	gelatin	—	Span 80	solid	o/w
2	OA2	octacosane	SDS	albumin	—	Span 80	liquid	o/w
2	OG2	octacosane	SDS	gelatin	—	Span 80	solid	o/w
2	SW2	squalane	SDS	—	—	Span 80	liquid	o/w
2	OW2	octacosane	SDS	—	—	Span 80	liquid	o/w
2	SA4	squalane	AOT	albumin	—	Span 80	oily	w/o?
2	SG4	squalane	AOT	gelatin	—	Span 80	oily	w/o?

^a^SDS = sodium dodecyl sulfate, AOT = sodium bis(2-ethylhexyl) sulfosuccinate (Aerosol-OT). ^b^Span 80 = sorbitan monostearate. ^c^o/w = oil-in-water emulsion, w/o? = presumably water-in-oil emulsion.

In total, we prepared 12 synthetic "insect" emulsions that were structurally characterized by microscopic techniques such as bright field, fluorescence and cryo-scanning electron microscopy (cryo-SEM) and laser diffraction particle size analysis. Parameters of particular interest were their phase volume ratios and droplet-size distributions. Their adhesive and frictional performances were determined by nanotribometric measurements. We thus aimed at clarifying the influence of the various chemical compounds such as lipids, proteins and carbohydrates on the tribometric and rheological properties of the emulsions. Statistical correlation analyses aided the evaluation of the interrelationships between the structural, chemical and performance attributes of the emulsions.

## Results

### Structural characterization of synthetic emulsions

**Figure 1:**
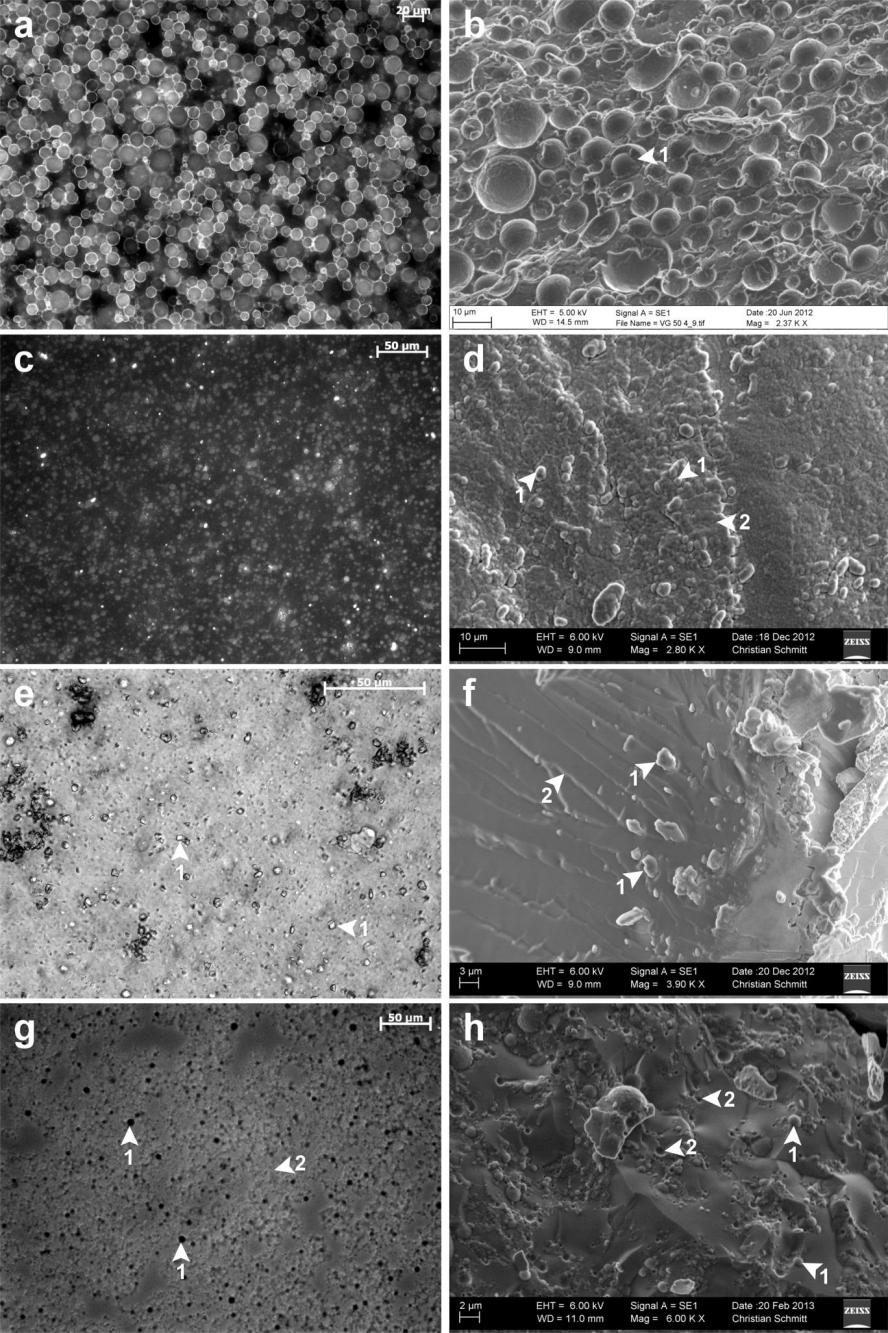
Droplet distribution of selected emulsions of the first and second generation. Left side: appearance of the emulsions under bright field light or fluorescence microscopy; right side: appearance of the emulsions under cryo-SEM. (a) Densely distributed fluorescent lipid droplets of emulsion VG50 (Sudan-III-stained emulsion was examined under light excitation of 300–400 nm). The fluorescent droplets are indicative of the o/w characteristic of this emulsion, because these fluorescent oily droplets form the inner phase. The outer hydrophilic phase is reduced because of evaporation. (b) Embedded, densely distributed droplets of emulsion VG50 surrounded by a slightly textured boundary layer (arrow 1). The emulsion has a droplet size range of about <1–50 μm, whereas most of the droplets lie between 5–15 µm. (c) Densely distributed fluorescent lipid droplets of the emulsion SG2 (Sudan-III-stained emulsion was examined under light excitation of 530–560 nm). The fluorescent droplets are indicative of the o/w characteristic of emulsion SG2, because these fluorescent oily droplets form the inner phase. (d) Distributed droplets (1) of emulsion SG2 embedded in an outer phase (2) with a rough appearance having a flaked structure. Main size range of round- to oval-shaped droplets is about <1–5 µm. (e) Loosely scattered and polyhedral droplets (1) of emulsion OA2 by light microscopy. The emulsion was examined under bright field after dilution with water. The lipid phase octacosane (1) consisted of solid polyhedral fragments causing aggregation. (f) Polyhedral fragments (1) of emulsion OA2 embedded in a lamellar-layered outer phase (2). Main size range of fragments is about <1–3 µm. (g) Non-fluorescent droplets (1) of emulsion SA4 embedded in a fluorescent outer phase (2) (the Sudan-III-stained emulsion was examined under light excitation of 530–560 nm). The non-fluorescent droplets in combination with the fluorescent outer phase are indicative of a w/o emulsion. (h) Smooth emulsion surface of SA4 consisting of both droplets (1) and cavities (2). The cavities (<1–2 µm) seem to represent the remains of evaporated water droplets following the sublimation step, whereas the unaffected droplets probably represent those that have not fully evaporated. Main size range of visible droplets is about 1–4 µm.

Table S1 (in Supporting Information File 1) summarizes the structural parameters of each emulsion as revealed by bright field/fluorescence microscopy and cryo-scanning electron microscopy (SEM). In general, the droplet sizes determined by the various methods, such as bright field/fluorescence microscopy and cryo-SEM, were in good correspondence, except for the larger size range of droplets as shown by the bright field/fluorescence microscopic images in comparison with those by cryo-SEM. The microscopic analyses revealed that the first generation provided a broader droplet size range with extremely large individual droplets leading to platykurtic kurtosis (Supporting Information File 1, Table S1). In contrast, most second generation emulsions showed narrower droplet size ranges, as indicated by their almost mesokurtic or leptokurtic distributions (Supporting Information File 1, Table S1). Both the emulsions within the second generation (SW2, OW2) without proteins possessed a narrower droplet size range compared with the emulsions having protein additions. Furthermore, all emulsion of both generations, except OA2 and OG2, had an almost symmetrical to positive skewness in common (Supporting Information File 1, Table S1). With regard to the rigidity of the emulsions at room temperature, the emulsions of the first generation were either solid or soft, whereas those of the second generation showed an aqueous or oily consistence (except for SG2 and OG2, which also exhibited a solid appearance) (Table 1; Supporting Information File 1, Table S1). In contrast to all other emulsions, those based on octacosane featured polyhedral droplets, which had a crystal-like appearance (Figure 1e,f; Supporting Information File 1, Figure S1a,b,f). In addition, in two out of three cases, the presence of octacosane led to unstable phases and to the formation of clumps within these emulsions (Supporting Information File 1, Table S1). Within emulsion SA2 (Supporting Information File 1, Table S1), the aqueous and the oily phase rapidly separated. The analysis of the emulsions of both generations by fluorescence microscopy revealed that all emulsions, except SA4 and SG4, displayed fluorescent droplets only thereby indicating the presence of oil-in-water (o/w) emulsions (Figure 1a,c; Supporting Information File 1, Table S1; Figure S2a,e,g). Vice versa, the two emulsions SA4 and SG4 possessed a fluorescent outer phase surrounding non-fluorescent droplets thus indicating the presence of water-in-oil (w/o) emulsions (Figure 1g; Supporting Information File 1, Table S1; Figure S1g). Against all expectations, the cyro-SEM images of SA4 and SG4 showed visible droplets within the outer lipid phase (Figure 1h; Supporting Information File 1, Figure S1h). These droplets possibly represented the remnants of water droplets that had not as yet fully evaporated. The dilution of both these emulsions with the assumed outer phase squalane was not possible and merely led to floating emulsion fragments within the squalane phase. Dilution with water was possible in SA4 only (Supporting Information File 1, Table S1).

### Rheological characterization of selected emulsions

**Figure 2:**
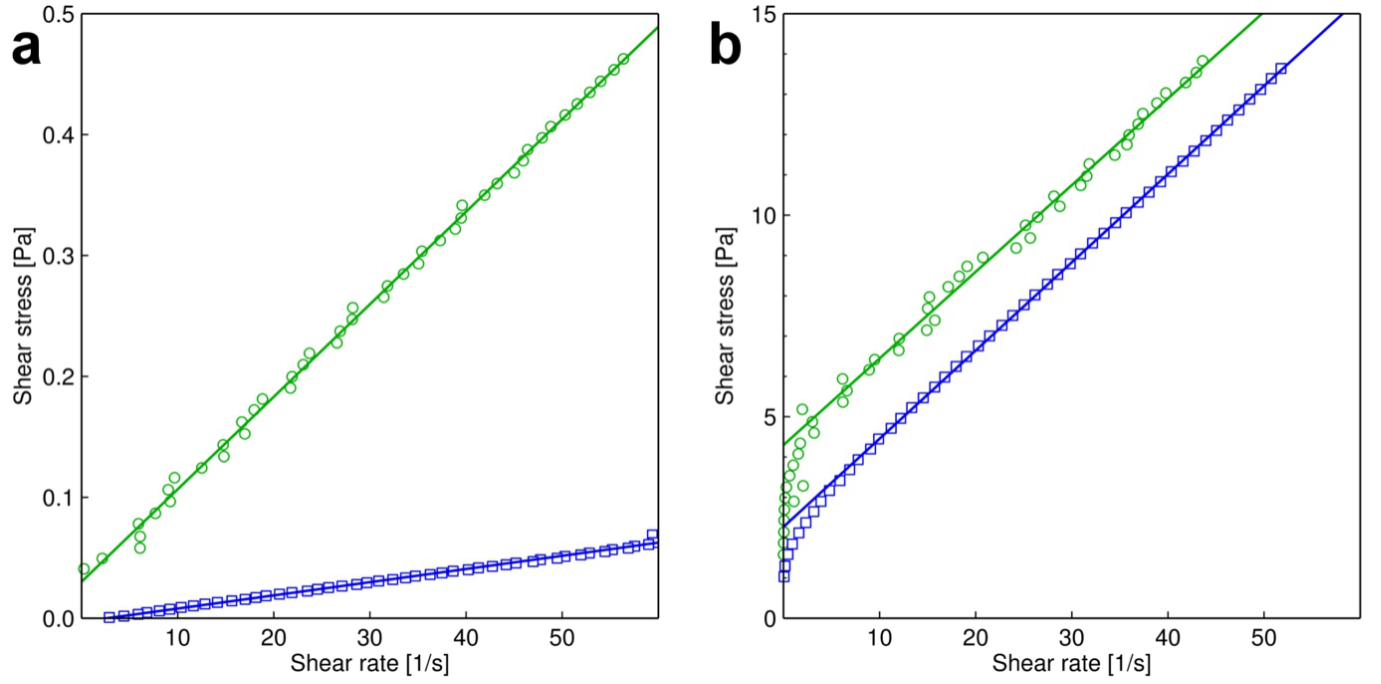
Rheological characterization of selected emulsions obtained by using plate-plate rheology within a shear rate range up to 60 s^−1^. (a) Green circles: emulsion SG2, blue squares: emulsion SA2, both measured at 40 °C. (b) Green circles: emulsion SG4, blue squares: emulsion SA4, both measured at 25 °C.

The rheological behaviour of selected emulsions was characterized by using the plate–plate geometry of the rheometer. In order to obtain information concerning the behaviour at different shear rates, they were increased from 0 up to 60 s^−1^. For this analysis, we examined the emulsions consisting of squalane and the proteins gelatin and albumin, respectively. We propose that a yield point is required for a locomotion adhesive to prevent the sliding of a non-moving insect from a vertical smooth surface. Therefore, the rheological properties were measured only for formulations that showed pronounced non-Newtonian flow behaviour by qualitative observation, a prerequisite for a rheological yield point. This was the case for the emulsions based on squalane and proteins. As a further structural difference, depending on their chemical composition, these emulsions showed oil-in-water (SA2 and SG2) or water-in-oil (SA4 and SG4) morphologies. As SG2 has a solid character at room temperature, the chemically related pair SA2 and SG2 was measured at 40 °C, whereas the pair SA4 and SG4 was measured at 25 °C. As the general rheological behaviour of the emulsions was to be evaluated (and not the exact viscosities), this procedure was acceptable. The measurements are shown in Figure 2. The emulsions SG2, SA4 and SG4 behaved as Bingham fluids showing yield stresses of 0.04 Pa, 2.5 Pa and 4 Pa. In contrast, SA2 behaved as a normal Newtonian fluid. In principle, the viscosities of the emulsions could be predicted from available models using the viscosity, the volume fractions and the droplet sizes of their components [33–34]. However, as some of the applied components (e.g., proteins) have an amphiphilic character, the prepared emulsions show a rather complicated morphology. In addition, according to their non-Newtonian rheological behaviour, such a calculation would be complex and beyond the purpose of this article.

### Tribological characterization of emulsions

#### Adhesion

**Figure 3:**
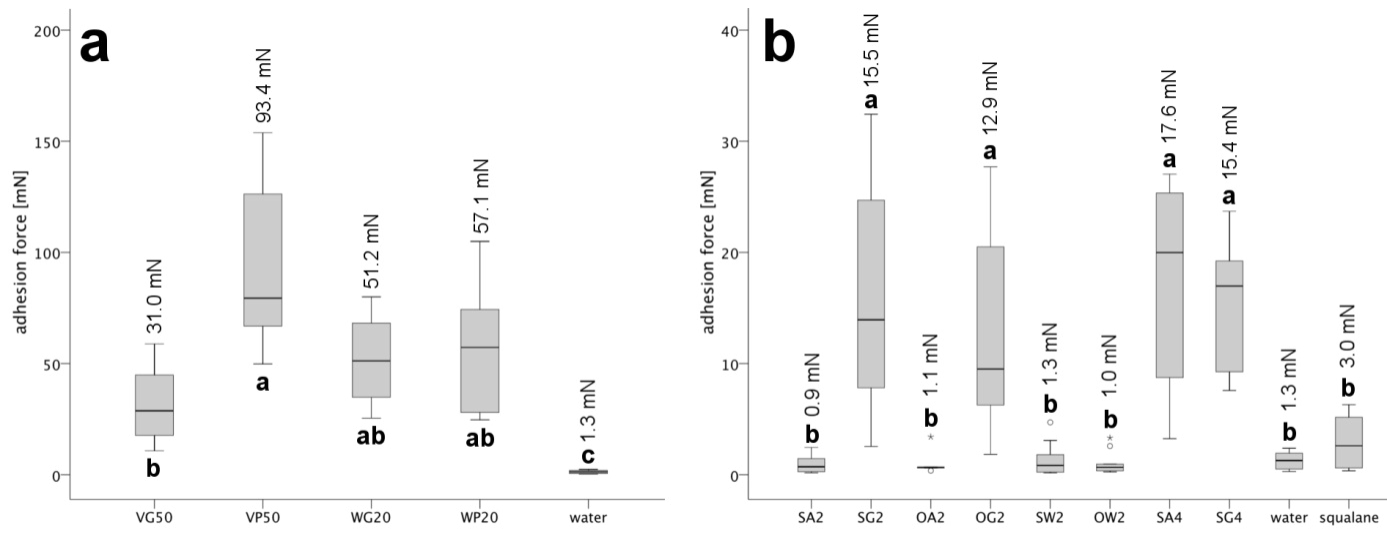
Box-plots of the adhesion forces of both generations of emulsions. The statistical comparison of the adhesion refers to the common logarithmized adhesion forces. Significant differences between the emulsion of logarithmized values (Supporting Information File 1, Tables S3 and S4) are indicated by different small letters (Kruskal–Wallis ANOVA followed by Kruskal–Wallis post hoc multiple comparisons). The measured values refer to a surface area of the silicium wafer of 6.45 mm^2^ and a normal load of 3.3 mN. The value above each box depicts the arithmetic mean of its adhesion measurements as reported in Supporting Information File 1, Table S2. (a) Adhesion forces of the first generation (Supporting Information File 1, Table S2). All four emulsions show similar adhesion forces, whereby the combination of petrolatum and poly(vinyl alcohol) solution in VP50 generated the highest forces. (b) Adhesion forces of the second generation (Supporting Information File 1, Table S2). The addition of gelatin gives stronger adhesion forces in the emulsions SG2, OG2 and SG4. The asterisk and circles above and below the boxes categorize outliers (asterisk) and extreme values (circle). Outliers are defined as values showing a distance of 1.5–3 times of the box height from the box border (25% and 75% quantile), whereas the distance of extreme values is larger than 3 times the box height.

The first generation of emulsions showed a significantly stronger adhesion than the second (Mann–Whitney-U test; *p* < 0.001; *N* = 12), i.e., more than twice as high an adhesion force (Figure 3; Supporting Information File 1, Table S2). Within the first generation, the adhesion of all emulsions, except VG50 and VP50, did not significantly differ from each other (Figure 3a; Supporting Information File 1, Table S3). However, emulsion VP50 exhibited the clear tendency of having the highest adhesion force, whereas emulsion VG50 showed the lowest adhesion force (Supporting Information File 1, Table S2). Moreover, all four emulsions revealed significantly higher adhesion compared with the control water (Figure 3a; Supporting Information File 1, Tables S2 and S3). Among the second generation, the four emulsions SG2, OG2, SA4 and SG4 (with average adhesion values between 13 and 17 mN) showed significantly stronger adhesion than the other emulsions of the second generation (Figure 3b; Supporting Information File 1, Tables S2 and S4). The remaining emulsions SA2, OA2, SW2 and OW2 and the controls water and squalane revealed no significant differences between one another (Figure 3b; Supporting Information File 1, Table S4). The adhesion force of these emulsions and water was between 0.9 and 1.34 mN (Supporting Information File 1, Table S2). Compared with these emulsions, the hydrophobic control squalane had slightly stronger adhesive abilities (3 mN) (Supporting Information File 1, Table S2).

#### Friction

**Figure 4:**
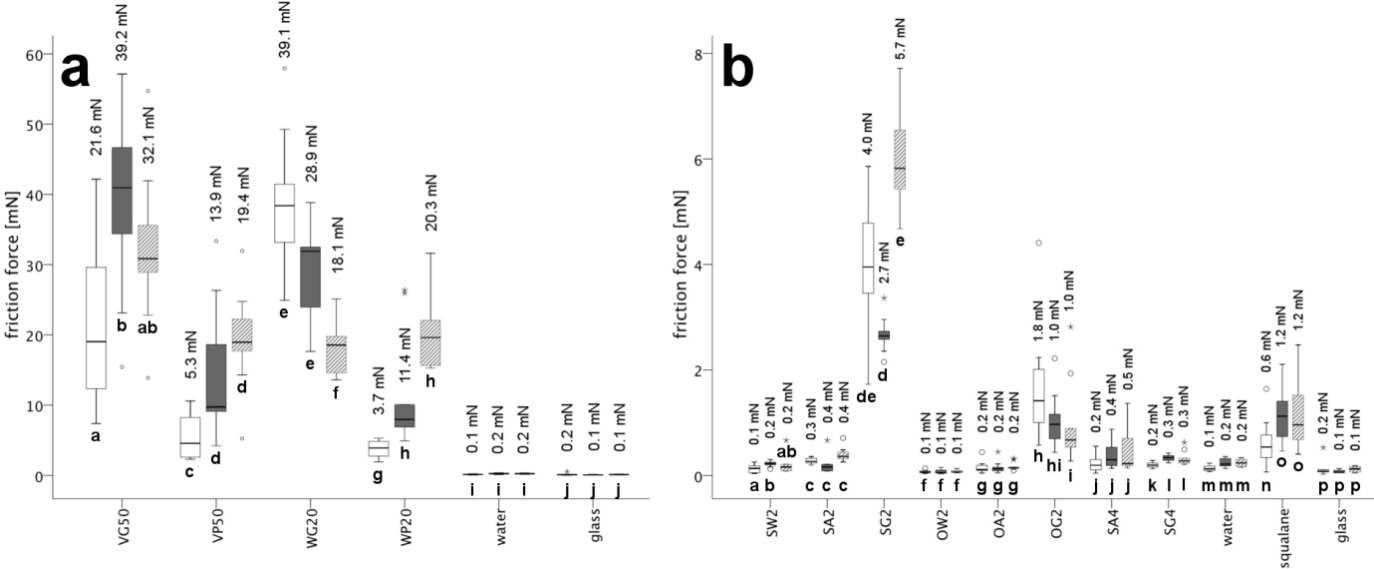
Box-plots of the friction forces of the emulsions of both emulsion generations at speeds of 50 (white), 200 (dark grey) and 500 (grey striped) µm s^−1^. The asterisk and circles above and below the boxes categorize outliers (asterisk) and extreme values (circle). Outliers are defined as values showing a distance of 1.5–3 times the box height from the box border (25% and 75% quantile), whereas the distance of extreme values is larger than 3 times of the box height. Significant differences between the three different velocities within an emulsion of logarithmized values (Supporting Information File 1, Tables S11 and S12) are indicated by different small letters (Friedman test followed by Friedman post hoc multiple comparisons). The statistical comparison of the friction within each emulsion refers to common logarithmized friction forces. The measured values refer to a surface area of the silicium wafer of 6.45 mm^2^ and a normal load of 0.6 mN. The value above each box depicts the arithmetic mean of its friction measurements as reported in Supporting Information File 1, Table S2. (a) Friction forces of the first generation (Supporting Information File 1, Table S2). The three emulsions VG50, VP50 and WP20 show an increase in the friction force with increasing speed, whereas the frictional performance of WG20 decreased with increasing speed. (b) Friction forces of the second generation (Supporting Information File 1, Table S2). The velocity only influences the emulsions SG2 and OG2 and the control group squalane, whereas the frictional performance of the remaining emulsions does not change with speeds.

First generation of emulsions: For the slowest speed (50 µm s^−1^), emulsion WG20 showed the highest friction force (39 mN), whereas at the higher velocities (200 and 500 µm s^−1^), emulsion VG50 gave the highest friction performance (39 mN and 32 mN, respectively) (Figure 4a; Supporting Information File 1, Table S2). Whereas the friction force increased with increasing sliding speed in VG50, VP50 and WP20, the opposite was the case in WG20 (Figure 4a, Supporting Information File 1, Tables S2 and S11). In addition, the friction curves of emulsion VG50 showed a stick and slip pattern (not shown), especially at 50 µm s^−1^; this was caused by the short-term sticking of the emulsion surface to the wafer alternating with a sudden tearing of the adhered surface once the maximum shear force was exceeded. For 50 and 200 µm s^−1^, emulsions VP50 and WP20 showed the lowest friction forces (Figure 4a; Supporting Information File 1, Table S2). Based on the statistical analysis, the emulsions based on poly(vinyl alcohol), namely VP50 and WP20, did not significantly differ from each other for all three speeds (Supporting Information File 1, Tables S5, S6 and S7). The same holds true for the two emulsions based on gelatin, namely VG50 and WG20, for the speeds 50 and 200 µm s^−1^ (Supporting Information File 1, Tables S5 and S6). Compared with the two controls of glass and water, emulsions VG50, VP50, WG20 and WP20 always gave significantly higher friction values (Supporting Information File 1, Tables S5, S6 and S7).

Second generation of emulsion: Overall, the friction performance of these emulsions was significantly lower compared with that of the first generation (Mann–Whitney-U test; *p* < 0.001; *N* = 12). For all the three speeds, the friction values of emulsions SA2, OA2, SW2, OW2, SA4 and SG4 were relatively low (similar to the controls water and glass) amounting from 0.1 to 0.5 mN (Supporting Information File 1, Table S2; the lowest values being attained in the protein-free emulsions SW2 and OW2). Among these emulsions, the friction forces of emulsions SA2, SA4 and SG4 were significantly higher than that of OW2 (for all velocities) and of OA2 and SW2 (for some velocities) (Figure 4b; Supporting Information File 1, Tables S2 and S8–S10), whereas the others showed no statistically confirmed differences among each other (Supporting Information File 1, Tables S8–S10). At 50 µm s^−1^, emulsion OG2 showed a significantly higher friction ability (arithmetic mean: 1.8 mN) than the previously mentioned emulsions, namely SA2, OA2, SW2, OW2, SA4 and SG4. With increasing velocity, the difference among OG2 and the other emulsions decreased (Figure 4b; Supporting Information File 1, Tables S8–S10). The highest friction forces within the second generation were measured for SG2 in a range of 2.7–5.7 mN for all three speeds (Supporting Information File 1, Table S2). Because of the high friction values of emulsion SG2, its difference was significant in comparison with all second generation emulsions, water and glass, except for OG2 and the control squalane (Supporting Information File 1, Tables S8–10). The third control group squalane showed slightly, but significantly, higher friction values (arithmetic means: 0.6–1.2 mN) than emulsions OA2, SW2, OW2, SA4 and SG4 and the two controls water and glass (Figure 4b; Supporting Information File 1, Tables S2 and S8–S10).

Whereas emulsions SA2, OA2, OW2 and SA4 and the two controls water and glass showed no dependence on the sliding velocity, this was indeed the case for emulsions SG2, OG2 and the control group squalane (Figure 4b; Supporting Information File 1, Table S12). The friction values of SW2 and SG4 varied only slightly with increasing speed (Supporting Information File 1, Tables S2 and S12). Similar to squalane, the friction forces of SG2 increased with increasing speed, but initially the friction force dropped, although not significantly, over the course of 50 to 200 µm s^−1^ (Figure 4b; Supporting Information File 1, Tables S2 and S12). The friction behaviour of emulsion OG2 was similar to that of the first generation emulsion WG20 (Figure 4a), showing a significant decrease in friction force with increasing sliding speed (Figure 4b; Supporting Information File 1, Tables S2 and S12).

### Statistical relationships between structural, chemical and tribological parameters

Relationship between chemical composition and structure of the emulsions (Supporting Information File 1, Table S13): Generally, all the structural parameters that were related to the droplet size of an emulsion (25–90% quantile, arithmetic mean, median, mode, standard deviation (Supporting Information File 1, Table S1)) showed a positive statistical correlation to the chemical compounds Vaseline, glycerine and SDS (sodium dodecyl sulfate), occurring mostly within the first generation of emulsions. The non-ionic surfactant Span 80 and the ionic surfactant AOT (sodium bis(2-ethylhexyl) sulfosuccinate) significantly influenced the droplet size of the 10% quantile and the phase volume. The excess kurtosis, representing the distribution of the droplet sizes, only correlated with the hydrophobic compound octacosane, which was used for the second generation emulsions OA2, OG2 and OW2.

Relationship between structure and adhesive/frictional performance of the emulsions (Supporting Information File 1, Table S14): The droplet size within the range of the 50–90% quantile and the droplet-size-dependent parameters (arithmetic mean, median, standard deviation) correlated positively with both the adhesive and the frictional performance, i.e., emulsions with higher droplet sizes showed higher adhesion and friction forces. The excess kurtosis correlated negatively to the friction ability and to the adhesion by trend, showing increased frictional and adhesive performances attributable to more balanced droplet-size distributions. From our correlations between the emulsion structure and its chemical composition (Supporting Information File 1, Table S13), we infer that the excess kurtosis is only influenced by the presence of the hydrophobic compound octacosane as used in emulsions OA2, OG2 and OW2. Moreover, the phase volume ratio showed no correlation related to the frictional performance.

Relationship between chemical composition and adhesive/frictional performance of the emulsion (Supporting Information File 1, Table S15): The compounds Vaseline, microcrystalline wax, poly(vinyl alcohol) (only at the highest sliding speed of 500 µm s^−1^), glycerine, gelatin and sodium dodecyl sulphate (SDS) showed a positive significant relationship with friction. In addition, friction was also negatively affected by the non-ionic surfactant Span 80 at an almost significant level. In contrast, adhesive performance correlated positively with poly(vinyl alcohol) and the ionic surfactant SDS, whereas the chemical compounds Vaseline, microcrystalline wax and gelatin influenced adhesion only by trend.

## Discussion

In contrast to bioinspired materials based on microstructured surfaces [35–36], the molecular biomimetics of the adhesive liquids (adhesives) involved in biological adhesive systems remains in its infancy [37–38]. Emulsion-based glues are widely spread in technology and are deployed not only in casein glues, but also in releasable contact adhesives such as tapes, patches and labels. However, in all known technical systems, the emulsion coalesces rapidly after application, forming a more or less homogeneous organic bond line.

Insect adhesive emulsions combine polar and non-polar components, and this combination might be decisive for the achievement of functional properties that are of high technical relevance, such as (i) versatility towards polar and non-polar surfaces, (ii) reversibility of the adhesive contact and (iii) robustness towards contamination and exsiccation.

Basing our present contribution on previous chemical analyses of insect tarsal adhesives [4,15–18,21], we have prepared heterogeneous synthetic emulsions mimicking the polar/non-polar principle, analysed their microscopical structure, and tested their adhesive, frictional and rheological properties. In total, we have prepared and tested 12 different biomimetic "insect adhesives" and, depending on their composition, have been able to attain a broad spectrum of micromechanical properties (cf. Figures 2–4). This shows that synthetic heterogeneous adhesive emulsions can, in principle, be adjusted by varying their chemical composition in such a way that they are able to mimic certain rheological and tribological properties of the biological role model and by their having various consistencies (cf. Supporting Information File 1, Table S1). With regard to their chemical composition, other than of water and hydrocarbons, we have used amphiphilic compounds selected on the basis of their resemblance to such compounds in nature. Moreover, in addition to proteins, these are amphiphilic polymers (namely poly(vinyl alcohol)) and tensides (namely Span 80), both mimicking fatty acids and carbohydrate compounds in natural role models.

### Structure

The prepared emulsions varied in their consistency from solid rubber-like, over soft elastic, to fluid (watery or oily). In general, the use of gelatin as the protein component (VG50, WG20, SG2, OG2) made the emulsions solid and rubber-like, because of the gel-like consistency of gelatin at room temperature. The only exception was the presumed water-in-oil (w/o) emulsion SG4. Despite its large amount of gelatin, the addition of squalane (fluid at room temperature) together with the ionic emulsifier AOT kept this emulsion in an oily state. Similar to gelatin, the addition of high-melting microcrystalline wax (WG20, WP20) led to a solid (rubber- to brittle-like) consistency, which, on the other hand, could be softened by the addition of poly(vinyl alcohol) (VP50). The remaining emulsions consistently showed a fluid consistence, which could be mostly ascribed to the use of squalane (if combined with albumin or gelatin at low concentration or if the protein component was omitted completely).

Most of our prepared synthetic emulsions represented oil-in-water (o/w) emulsions. Two emulsions (SA4 and SG4) that were stabilized with the hydrophilic emulsifier AOT were water-in-oil (w/o) emulsions, although the true nature, especially of SG4, remained ambiguous. With droplet sizes >100 nm, all the emulsions belonged to the common type of macroemulsions, although the emulsions can readily be classified according to differences with regard to their droplet-size distributions (Supporting Information File 1, Table S1). The emulsions of the first generation generally showed broader (more platykurtic) droplet size ranges with higher standard deviations compared with the second generation. This was certainly the result of the employment of less defined components such as petrolatum or waxes (consisting of hydrocarbons of various lengths and degrees of branching) in the lipophilic fraction of the first generation of emulsion. The use of clearly defined hydrocarbons (octacosane or squalane) together with the employment of the additional surfactant Span 80 in the second generation of emulsion in most cases resulted in narrower (almost mesokurtic or leptokurtic) distributions (showing lower standard deviations). Among the second generation emulsions, both the protein-free emulsions (SW2, OW2) clearly showed smaller droplet sizes compared with the protein-containing emulsions. This is probably caused by the presence of the much smaller amphiphilic Span 80 molecules, which are able to form common emulsion drops. In contrast, the large protein molecules (even in the presence of the smaller Span 80 emulsifier molecules) need a much larger area once they form the interphase between the aqueous and the oil phase. Most likely, because of their large molecular size, the proteins are even able to form bridges between smaller droplets previously emulsified by the Span 80 molecules. For insect adhesive emulsions, few data are available on the droplet sizes of the inner phase. They are in the range between 100 nanometers and several micrometers [14,20–21] and thus fit well in the range of almost all of our second generation emulsions (cf. Supporting Information File 1, Table S1).

Interestingly, in the (second generation) octacosane-based oil-in-water (o/w) emulsions, the octacosane forms solid crystal-like particles that are suspended in the outer matrix; this is certainly attributable to the high-melting temperature of octacosane (>60 °C). Such colloidal suspension-like behaviour corresponds well to the assumed nature of the outer lipid layer of the insect cuticle [4,28,39–40] and can also be assumed for insect tarsal adhesives being mere derivatives of the outer free lipid layer of the general body cuticle [41–43]. Such mixtures of high-melting straight *n*-alkanes with low-melting alkenes or methyl-branched alkanes keep the suspensions in a semi-solid condition over a broad range of temperatures. The in situ phase differentiation of alkanes and alkenes/methyl-branched alkanes at ambient temperatures, forming a colloid suspension of solid wax crystals within a liquid matrix [28], might induce rate-dependent viscosity changes caused by non-Newtonian shear strains. Non-Newtonian viscosity shifts are also common properties of emulsions [24–25], which consist of an aqueous phase dispersed in an oily continuous phase or vice versa. Hence, we wished to test our synthetic "insect adhesives" for such shear thinning properties in shear tests and to compare their adhesive and frictional properties with a nanotribometer.

### Rheology

Previously, in insect tarsal adhesive systems, any kind of liquid functioning as a reversible adhesive during movement was thought to exhibit a non-Newtonian Bingham-like rheological yield point for the production of sufficient static friction in order to prevent sliding on vertical substrates when the insect was at rest [14]. Such flow behaviour is actually considered a general characteristic of emulsions, whereupon the yield points are differently pronounced [29]. We subjected four of our 12 synthetic emulsions to plate-plate rheology. To different extents, emulsions SG2 (0.04 Pa), SA4 (2.5 Pa) and SG4 (4 Pa) show Bingham-like behavior, whereas the rheology of SA2 is consistent with a Newtonian fluid. The latter o/w emulsion has a watery consistence and shows a comparatively low phase volume ratio (0.06) with only small and widely scattered oil droplets, which suggests that in this case the continuous water phase outweighs any influence of the dispersed oil phase. The rheological comparison between the emulsions SG2 and SA2 suggests that albumin has a lower ability to form emulsions with yield points than does gelatin. This behaviour is also pronounced in both the water-in-oil (w/o) emulsions SA4 and SG4 (cf. Figure 2b). Gelatin is known to form gel-like structures, which is probably the reason for more pronounced yield points in the gelatin-based emulsions. In the w/o emulsions, the polymeric protein components probably form hydrophilic drops within the hydrophobic phase. To initiate flow, these drops must be deformed, probably leading to the observed yield point.

### Adhesion

In terms of their adhesive performance, our prepared emulsions showed considerable differences, ranging from 1–93 mN. Compared with the second generation, the first generation emulsions were much more adhesive (31–93 mN); this is attributable to their highly viscous components, i.e., wax and petrolatum (with their long chain lengths), gelatin and poly(vinyl alcohol) (Supporting Information File 1, Table S1). Because of its slight tackiness, poly(vinyl alcohol) is actually employed as component of technical glues [44]. In the second generation emulsions, we attained much lower values of adhesiveness, ranging between 1–18 mN. Whereas the gelatin-containing emulsions of this generation still showed comparatively high stickiness (13–15 mN), the adhesive performance was drastically reduced in the emulsions that contained albumin as the protein component or that did not contain any protein at all (0.9–1.3 mN). In this respect, they resembled the controls of water and pure squalane (although the adhesiveness of pure squalane tended to be slightly higher). Although containing the protein component albumin instead of gelatin, the adhesiveness of the presumed water-in-oil (w/o) emulsion SA4 was as large as in the gelatin-containing emulsion SG4. This indicates that both proteins are mainly present in the aqueous phase of the emulsion surrounded by the same oil phase, the latter forming the contact with the substrate and thus determining the adhesive performance. The similar adhesive performance of both these emulsions further supports the view that both these emulsions are actually water-in-oil (w/o) emulsions. Both albumin and gelatin are proteins that have been used in artificial adhesives but they were applied mainly in the past.

Although, the adhesive structures of insects are deployed in a reversible manner, depending on the biological context (e.g., locomotion versus prey-capture), the required forces can vary considerably [4,45]. Such different demands are well reflected by the different values of adhesiveness of our synthetic emulsions. Because of their small quantities, only a few attempts have been undertaken, to date, to determine the adhesive stress of insect tarsal adhesives in isolation from their underlying cuticle [5,46]. Moreover, to our knowledge, no attempts have as yet been undertaken to quantify the portion that the adhesive secretion contributes to the total adhesive performance of an intact insect tarsus with respect to diverse surface regimes. For effective locomotion, the adhesive and/or cohesive forces must be held at a moderate level to enable the rapid and effortless re-release of the tarsal surface after contact formation. In the wet adhesive systems of insect tarsi, adhesive stresses (tenacities) measured without shear range between 1.1 and 7 kPa [47]. These values have been determined in intact tarsi and are the result of the combined properties of the viscoelastic tarsus cuticle together with the overlying adhesive secretion. Related to the surface area of the used measuring heads, the adhesive stresses of our prepared synthetic emulsions are in the range of 7.9–14.4 (first generation) and 0.2–2.8 kPa (second generation). From this perspective, the adhesive strength of our first generation of emulsions appears over-large in terms of the necessity to create reversible attachment structures mimicking insect attachment structures employed in locomotion (this holds true all the more, since we have tested the adhesive strength of the fluids between two rigid plates that show no distortion during their separation). During the detachment process, excessively sticky tarsal insect adhesives might actually transfer their viscous dissipation to the viscoelastic cuticle, largely hampering tarsal release. Indeed, recent empirical and theoretical analyses in stick insects predict that the adhesive strength and viscosity of the tarsal secretion should be rather low, thereby decreasing viscous dissipation during tarsal retraction [48]. From this perspective, our four second generation emulsions SA2, OA2, SW2 and OW2 (all combining the hydrocarbons squalane or octacosane with the protein albumin or leaving out the protein component) might come closest to this demand. Their fluid consistency might also help to compensate for the roughness of microstructured surfaces to maximize contact. During the separation of the wafer from the glass surface, their failure was cohesive, i.e., the fluid thread broke somewhere in its middle. Many insect tarsal adhesives might behave similarly, with the tarsal adhesive possibly being cohesively adjusted in a way that minimizes material loss.

From a biomimetic perspective, four of the second generation emulsions that combine considerably increased (though still moderate) adhesive strength (about 2 kPa) with a semi-solid (rubber-like) (SG2, OG2) or oily (SA4, SG4) appearance might be of special interest. Indeed, the hydrocarbon pattern established by recent chemical analyses suggests a similar semi-solid (grease-like) consistency of insect adhesives [15–17] and such a property would consolidate several functions in the context of effective locomotion (and possibly technical applications) such as Bingham-like slip resistance, tarsal releasability, desiccation resistance, mechanical compliance and protection from abrasive damage. Tarsal releasability might be brought by since the tarsal adhesive secretion actually acting as a kind of "release-layer" (due to its reduced wetting ability according to its semi-solid consistence), minimizing the viscous dissipation of both the adhesive liquid and the viscoelastic pad material during detachment [48]. On the contrary, adhesive systems employed in prey-capture, such as the sticky labial pads (paraglossae) of *Stenus* species (Coleoptera, Staphylinidae), seem largely to depend on such energy-dissipating effects of both the viscous adhesive and the highly elastic (resilin-containing) pad material in order to attain sufficiently high adhesion ([49] and Figure 6 in [50]). Hence, a comparison of both the chemical composition of the glue and the viscoelastic behaviour of the pad cuticle involved in this system with those deployed in tarsal attachment would be of interest.

### Friction

In dynamic attachment situations such as adhesive fluid-mediated insect locomotion, attachment forces are largely determined by the viscosity of the fluid, because of its influence on the shear stress generated during friction. High viscosity fluids should therefore be avoided during locomotion, because this would be counterproductive for the detachment process. In our experiments, in order to ensure that the shear stress of the bulk emulsions was assessed in a hydrostatic or hydrodynamic sliding regime (preventing solid–solid contact between the sliding surfaces or solidification processes that might occur in confined liquids), the load employed for measuring friction was set considerably lower than in the adhesion experiments, thereby maximizing film thickness. In accordance with theory that predicts lower friction with increasing film thickness [51–52], our measured shear stresses, i.e., the reported friction forces divided by the surface area of the used measuring heads, are comparatively low (0.6–7.3 kPa in the first and 0.009–0.9 kPa in the second generation emulsions). Being mainly determined by the viscosity of the emulsions, they are far from reaching the friction values measured in the isolated adhesive secretion of stick insects (lying in the range of about 100 kPa [5]) or intact insect tarsi (that can attain several hundred kPa [47,53–54]). Such differences are largely attributable to the different film thicknesses that have arisen during these experiments. Whereas, in thick fluid films, friction is largely determined by the viscosity of the liquid, in thin films, friction can be enhanced by processes such as (1) the formation of dry contacts by dewetting, (2) the solid-like behaviour of the liquid attributable to non-Newtonian properties, (3) the molecular ordering of the liquid at zones at which the film becomes thinner than a few monolayers and (4) the penetration of surface irregularities through the liquid film resulting in solid–solid contacts (cf. discussion in [55]). The influence of film thickness can also be seen in our experiments. Although we assume that in all of our experiments, we measured in the regimes of hydrostatic or hydrodynamic lubrication (cf. [56]), our nanotribometric experiments revealed much higher shear stresses than the plate-plate rheology. Whereas most experimental conditions were basically comparable in both these methods, they differed in the obtained layer film thickness (gap size). In the plate–plate-rheology, it amounted to 540 µm, which ensures the characterization of the emulsions as a bulk component. In contrast, in the nanotribometry experiments, the film thickness was 43 µm at maximum (probably lower according to the applied normal load), which ensured similar conditions as expected for insect tarsi during locomotion. Several mechanisms might influence the flow behaviour of liquids in confined spaces (e.g., [56–57]). In our nanotribometry experiments, at least the larger droplets of the emulsions are in the size range of the measurement gap applied, so that they might have become deformed or even finer dispersed under these conditions and this way changed the rheological behaviour of the emulsions compared to the properties of the bulk.

Friction was tested at three different sliding speeds to make the comparisons between the emulsions more reliable with respect to possible velocity dependencies of the friction performance. According to their (semi-)solid consistency (which is attributable to the higher viscosity of their constituents), our first generation emulsions showed a considerably higher friction performance, ranging from 4–93 mN (depending on the sliding speed) compared with the emulsions of the second generation and the squalane and water controls. Because of to their often rubber-like appearance, our measurements of these emulsions probably did not take place in the fluid friction regime but represent rubber friction [56]. Moreover, in such a regime, one expects an increase of friction with increasing sliding velocity [58–60] as has been shown by most of our first generation emulsions. In emulsion WG20, we established the opposite behaviour, i.e., the friction force decreased with increasing sliding velocity (Figure 4a). In this case, at the slowest sliding speed, we observed a clear stick–slip behaviour, which is known to produce especially high friction at local asperities but to decrease at higher sliding speeds [56,61].

The emulsions of the second generation show considerably lower friction forces that, depending on sliding velocity, range from 0.1–5.8 mN. Most of these emulsions show friction values as low as that of water, being even lower than the pure squalane used as a control. In the oil-in-water (o/w) emulsions, the phase volume ratio amounted to <1, so that their flow characteristics were largely dominated by the continuous watery phase. In combination with the use of the ionic surfactant SDS (which lowers the surface tension even further), this probably explains the low friction values of these emulsions. By contrast, two of the oil-in-water (o/w) emulsions (SG2, OG2) showed increased friction values; this can be ascribed to their semi-solid (rubber-like) appearance (because of their gelatin content) that was previously mentioned as explaining their increased adhesiveness (see section Discussion/Adhesion). Both the presumed water-in-oil (w/o) emulsions (SA4, SG4) show friction performances that are slightly increased towards both the water control and the above-mentioned oil-in-water (o/w) emulsions showing water-like low friction values. In these emulsions, the oily proportion is largely increased with respect to the water fraction, so that the lipid component squalane is able to take effect in enhancing the friction.

The friction values measured under the mediation of an emulsion almost always showed higher values than the friction obtained under dry conditions (i.e., direct sliding of the smooth wafer onto the smooth glass surface), suggesting a friction-enhancing effect of the fluid, even if its friction performance is similarly low to that of water (Figure 4b, Supporting Information File 1, Table S2). Under the dynamic situation of sliding friction in a soft tribology regime with elastomer-like tarsal adhesion structures (cf. [29]), this can be attributed to the contribution of the viscous forces of these fluids to friction.

## Conclusion

In the present contribution, we abstracted the polar/non-polar principle of emulsions to mimic the tarsal liquid that is secreted from insect feet in order to enhance tarsal attachment. One central property that we desired to achieve was the combination of reversible adhesion and easy release (peeling off) from the substrate. Whereas such a combination is difficult to attain with conventional adhesive systems, it is essential for insects for effective locomotion. Although many constructional adhesives (e.g., casein glues, adhesive tapes, medical patches and adhesive labels) are heterogeneous, their emulsions easily lose their structure after application resulting in a homogeneous bond line. The "polar/non-polar" principle involving lipid components has not as yet been accomplished in this context, although it might help to construct versatile adhesive systems that, for instance, make possible repeated and reversible contact and release without the degradation of the adhesive performance. Similar to the insect role model, such biofunctional adhesives might be combinable with microstructured adherents (e.g., technical polymer foams and sheets) in order to make use of possible synergisms between the structural component of the carrier (e.g., its viscoelasticity or the geometry of its surface) and the physico-chemical properties of the adhesive. The combination of emulsion-based adhesives and porous carrier materials should make possible the fine-tuning of bonding technological properties, especially in the range of low adhesive forces. Possible fields of application are initially non-sticky tapes whose adhesiveness can be activated by compressive stress, medical (with active pharmaceutical ingredients loadable and easily (free of pain) removable) patches, fluid-supplied medical patches for the treatment of burns and, with respect to their adhesiveness, controllable capillarity-based adhesion devices [62].

The adhesive strengths of our synthetic "insect emulsions" of the second generation lie well within the range of tenacities measured in intact insect tarsi. Considering their droplet sizes, they also seem to be structurally most similar to their biological role models. In our experiments, we measured the adhesive performance between two rigid (non-deformable) plates, whereas in tribological measurements of intact tarsi, one always measures the influence of the energy dissipation in the viscoelastic cuticle, which provides additional resistance towards separation. This suggests that natural insect tarsal adhesives actually show only low adhesive strengths that are in the range of a few kilopascal. Such properties have been produced especially in four of the emulsions of the second generation (SA2, OA2, SW2, OW2) by using the hydrocarbon components squalane (liquid at room temperature) or octacosane (solid at room temperature), the latter forming a colloidal lipid suspension. Whereas the adhesion and friction properties of these emulsions are similar to those of water, they probably show, because of their hydrocarbon components, additional functional properties of technical relevance such as improved resistance towards desiccation and contamination, and beneficial wetting properties towards both hydrophilic and hydrophobic surfaces.

The low adhesive strengths of natural insect tarsal adhesives are in strong contrast to the extremely high shear stresses measured in intact insect tarsi; these stresses can exceed adhesive tenacities by one to two orders of magnitude. We must assume that such discrepancies are the result of thin film thickness and boundary lubrication effects [56] in combination with the viscoelastic properties of pliable tarsal cuticles. In our nanotribometric friction experiments involving low normal loads and high liquid film thicknesses, we did not determine shear stresses under the conditions of such very thin liquid films in confined geometries, but rather assessed the viscosity of the bulk emulsion. As expected, these experiments revealed especially low shear stresses in the four emulsions of the second generation (SA2, OA2, SW2, OW2) that had previously shown low adhesive strengths. In addition, despite of their relatively high adhesiveness, the two presumed water-in-oil (w/o) emulsions SA4 and SG4 of the second generation exhibited similarly low shear stresses. Such behaviour (low shear stress at relatively high adhesive strength) might make possible the easy detachment of adhesive bonds by applying shear forces to the connected surfaces. Both these emulsions might actually resemble our biological role models most closely, since previous studies on the emulsion structure of insect tarsal adhesives have provided evidence that they are water-in-oil emulsions [14,23]. From this perspective, our technical emulsions might provide a clue as to the way that tarsal insect adhesives reconcile easy detachment at moderately high adhesive strength. In both these emulsions, our rheological experiments have established Bingham fluid-like shear thinning behaviour that shows an initial minimum "yield stress" before the emulsion start to flow [14].

In addition to such oily water-in-oil (w/o) emulsions, many insects [16–17] seem to exhibit (semi-)solid-like tarsal adhesives as achieved in all of our preparations of the first generation and two emulsions (SG2, OG2) of the second generation. In particular, in natural insect adhesives, the complex composition with fluidity enhancers (represented by branched and unsaturated hydrocarbons) adds to a semi-solid-like base. In addition, a melting point depression attributable to mixtures of diverse hydrocarbons might support such behaviour. In particular, OG2 comes close to the demand of low shear stress at moderately high adhesive strength that should facilitate detachment.

Our research suggests that technical systems inspired by emulsion-like insect adhesives might benefit from the possibility of adjusting their adhesive connections to current demands. In such devices, undesired detachment can be combined with easy removability, whereby the required attachment and detachment forces can be fine-tuned via polar and non-polar components and amphiphilic emulsifiers, their respective mixing ratios and the specific conditions under which the emulsions are prepared.

## Experimental

### Preparation and composition of synthetic emulsions

#### Preparation of synthetic first generation emulsions (VG50, VP50, WG20 and WP20)

The following solutions were prepared to form the desired emulsions:

- A gelatin/glycerine solution (20 wt %) was prepared by mixing 64.5 g gelatin (Sigma-Aldrich, Munich, Germany), 35.5 g glycerine (Ph. Eur., Sigma-Aldrich), 0.10 g sodium azide (as biocide, Sigma-Aldrich) with 400 mL deionized water. The obtained mixture was stirred at 40 °C until the gelatin had been completely dissolved.
- A poly(vinyl alcohol) solution (20 wt %) was obtained by the addition of 100 g poly(vinyl alcohol) (Mowiol 10-98, Ter Hell & Co. GmbH, Hamburg, Germany) and 0.10 g sodium azide to 400 mL deionized water. The obtained mixture was stirred at 80 °C for 2.5 h until the poly(vinyl alcohol) had been completely dissolved.

In the case of stained emulsions, the hydrophobic components microcrystalline wax (Sasolwax^®^ 1800, Sasol Germany GmbH, Hamburg, Germany) and Vaseline (Ph. Eur., Sigma-Aldrich) were mixed with 0.01 wt % Sudan III (Sigma-Aldrich) at 80 °C until complete dissolution of the dye.

The emulsions were created by mixing sodium dodecyl sulfate (SDS) (Sigma-Aldrich) with the poly(vinyl alcohol) or gelatin solution in a SpeedMixer^TM^ cup. After addition of the wax or Vaseline, the samples were homogenized in a SpeedMixer^TM^ (Hauschild & Co. KG, Hamm, Germany) for three times at 3500 rpm for 30 s. The compositions of the prepared emulsions are shown in Table 2.

**Table 2:** Composition of first generation emulsions.

emulsion name	hydrophilic solution	hydrophobic component	amount SDS

VG50	30 g poly(vinyl alcohol) solution	30 g Vaseline	172 mg
VP50	30 g gelatin solution	30 g Vaseline	172 mg
WG20	48 g poly(vinyl alcohol) solution	12 g Sasol wax	69 mg
WP20	48 g gelatin solution	12 g Sasol wax	69 mg

#### Preparation of synthetic second generation emulsions (SA2, SG2, OA2, OG2, SW2, OW2, SA4 and SG4)

A sodium azide solution was obtained by dissolving 80 mg sodium azide in 400 mL deionized water. A sodium azide/SDS solution was prepared by dissolving 80 mg sodium azide and 96 mg SDS in 400 mL deionized water.

In the case of stained emulsions, squalane (>95% technical grade, Sigma-Aldrich) and octacosane (99%, Sigma-Aldrich) were mixed with 1.5 ppm Sudan III until they had dissolved at room temperature and 80 °C, respectively.

The protein-containing emulsions were created by dissolving gelatin or albumin (Fraction V, >98%, Carl Roth GmbH & Co. KG, Karlsruhe, Germany) in 46 g of the prepared sodium azide/SDS solution. Albumin and gelatin were dissolved at room temperature and 40 °C, respectively. The hydrocarbon (squalane or octacosane) and the surfactant Span 80 (sorbitan monooleate, for synthesis, Carl Roth GmbH & Co. KG) were added to the solution. The mixture was homogenized by the impact of ultrasound (Bandelin Sonoplus, Bandelin electronic GmbH & Co. KG, Berlin, Germany) with the ultrasound transducer UW 2070, stepped standard horn SH 213G and sonotrode VS70T. During sonification with maximum ultrasound power, the temperature of the solution had to be controlled and kept between 60 and 65 °C. The sonification time did not exceed 1 min. The two protein-free emulsions SW2 and OW2 were prepared in the same way as the protein-containing emulsions, but without adding proteins. The compositions of the protein-containing and protein-free emulsions are shown in Table 3.

**Table 3:** Composition of second generation emulsions with and without protein content.

emulsion name	squalane	octacosane	Span 80	sodium azide/SDS solution	albumin	gelatin

SA2	1.98 g	—	0.84 g	46 g	3.18 g	—
SG2	1.98 g	—	0.84 g	46 g	—	3.18 g
OA2	—	1.98 g	0.84 g	46 g	3.18 g	—
OG2	—	1.98 g	0.84 g	46 g	—	3.18 g
SW2	1.98 g	—	0.84 g	46 g	—	—
OW2	—	1.98 g	0.84 g	46 g	—	—

The inverse (w/o) emulsions were prepared by dissolving albumin and gelatin in a sodium azide solution at room temperature and 40 °C, respectively. A second solution was prepared with squalane, Span 80 and AOT (sodium di(ethylhexyl)sulfosuccinate, Sigma-Aldrich) by continuous stirring. Both solutions were combined and homogenized by ultrasound (see conditions above). The compositions of the inverse emulsions are shown in Table 4.

**Table 4:** Composition of second generation inverse emulsions.

emulsion name	squalane	AOT	Span 80	sodium azide solution	albumin	gelatin

SA4	25.6 g	0.11 g	10.8 g	14.6 g	1.0 g	—
SG4	25.6 g	0.11 g	10.8 g	14.6 g	—	1.0 g

The general appearance of the prepared emulsions (cf. Supporting Information File 1, Table S1) of both the first and the second generation was evaluated by mechanically probing them with a spattle.

### Descriptive analysis of emulsion structure

#### Light optical and fluorescence microscopy

Microscopic assessment of the emulsions was performed by using an optical light microscope Zeiss Imager.Z1 (Carl Zeiss Microscopy GmbH, Jena, Germany). Both types of emulsion, water-in-oil (w/o) emulsions and oil-in-water (o/w) emulsions, were diluted with the respective outer phase prior to microscopic assessment. In the case of solid emulsions, a piece of the emulsion was picked and transferred into a microcentrifuge tube containing the respective outer phase. The mixture was liquefied by briefly being heated on a hot plate (Heidolph MR 3001 K; Heidolph Instruments GmbH, Schwabach, Germany). Some droplets of the diluted emulsions were placed on a microscope slide and carefully covered with a coverslip avoiding the entrapment of air bubbles.

The phase distribution of the emulsions was assessed by fluorescence microscopy of the stained emulsions. Fluorescent emulsions were formulated by dissolving Sudan III in the oil phase at a concentration of 0.1 % (w/w). Images were obtained with a fluorescence microscope (Zeiss Imager.Z1; Carl Zeiss Microscopy GmbH) by using an HBO 100 mercury lamp, the filter set 43 (excitation: 550/25, emission: 605/70) and the filter set 49 (excitation: 365, emission: 445/50). In both light and fluorescent microscopy, images were recorded with an AxiCam MRm camera (Carl Zeiss Microscopy GmbH) and Axiovison 4.7.1 software (Carl Zeiss Microscopy GmbH). For fluorescent microscopy, the oil phase was identified as the fluorescent phase, whereas the aqueous phase appeared dark.

#### Cryo scanning electron microscopy (cryo-SEM)

For this application, we mainly followed an already established protocol ("method 1" in [63]). In the case of solid emulsions, a small piece with dimensions 6.5 mm × 4.1 mm × 3.0 mm was cut out and glued into the hole of a copper carrier with Tissue-Tek^®^ O.C.T.TM (Sakura Finetek Europe B. V., Alphen aan den Rijn, The Netherlands). In the case of liquid emulsions, the emulsion was homogenized by an ultrasonic transducer UP200S (Hielscher Ultrasonics, Teltow, Germany) at an intensity of 85% for two minutes. After homogenization, the liquid was added in a hole of a copper carrier until the formation of a bulge on the surface of the carrier. We used the transfer unit/preparation chamber Emitech K1250X (Quorum Technologies, Laughton, UK) (the sublimation time amounted to 3–4 min at −95 °C to −80 °C) and the SEM Evo LS10 (Carl Zeiss Microscopy GmbH, Jena, Germany).

The resulting cryo-SEM images were analyzed by single measurements of about 20 randomly distributed droplets by using the digital image processing software AxioVision (v. 4.6.3, Carl Zeiss Microscopy GmbH).

#### Determination of type of emulsion

The fluorescent microscopical images were used to evaluate the type of emulsion, i.e., oil-in-water (o/w) versus water-in-oil (w/o). Emulsions with fluorescent droplets against a dark background were classified as oil-in-water (o/w) emulsions, because only the stained lipid phase possessed fluorescent properties. Vice versa, fluorescence shown by the outer phase only in combination with dark (non-fluorescent) droplets was indicative of a water-in-oil (w/o) emulsion.

#### Droplet-size distribution

The analysis of the droplet-size distribution was based on both the microscopic fluorescent and bright field images. To this aim, in Adobe^®^ Photoshop^®^ CS4 11.0, clear visible droplets within a selection of 1–3 fluorescent and bright field images (Supporting Information File 1, Figure S3a) were painted with an intense colour, i.e., green or red (Supporting Information File 1, Figure S3b). In ImageJ (W. Rasband, v. 1.47n), all the coloured droplets were marked by the "colour threshold tool" to convert them into a black and white image with clear black droplets. The largest and the smallest diameter of each droplet was measured by the function "analyze particles". Based on the arithmetic means of the smallest and the largest diameter of a droplet, the main size range of droplets within the light/fluorescence microscopy was defined by the range of the 15% and the 85% quantiles. Afterwards, the volume *V* of the measured droplets was calculated by Equation 1.

[1]
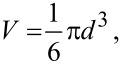


where *d* is the arithmetic mean of the smallest and largest diameter of a droplet. For a better overview, all the volumes were arranged in certain defined droplet-size groups. Therefore, a Microsoft EXCEL table with defined droplet-size ranges and equivalent volume ranges was created that served as a template to sort all the measured volumes. The percentage by volume (Equation 2) was calculated to illustrate the actual percentage of each group compared with the total volume of all the droplets together:

[2]
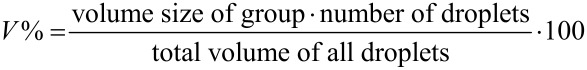


#### Determination of droplet sizes by Laser diffraction

The droplet-size distribution of the emulsions was determined by laser diffraction by using a Beckmann Coulter LS (Brea, CA, USA) equipped with a micro liquid module as the measurement cell. The applied detection range was 0.04–2000 µm and the optical model used for calculation of droplet-size distribution was Fraunhofer plus PIDS. For the measurements, the samples were diluted with deionized water.

#### Calculation of phase volume ratios

The phase volumes of the dispersed and continuous fractions of the prepared emulsions used as model adhesives were estimated by the applied composition. The volumes of the dispersed and the continuous phases were determined based on their densities (Supporting Information File 1, Table S16). The amphiphilic compounds AOT and SDS were not considered as they could not be assigned to one of the phases. These compounds formed instead the interphase [64], which had an unknown thickness and also contained unknown amounts of the hydrophilic and hydrophobic compounds. However, for the non-ionic surfactant Span 80, three possible distributions were assumed: (1) Span 80 fully dissolved in the hydrophilic fraction, (2) Span 80 fully dissolved in the hydrophobic fraction or (3) the fatty acid section of Span 80 dissolved in the hydrophobic fraction, whereas the sorbitan section dissolved in the hydrophilic fraction. The calculation of the phase volume of each emulsion was achieved by the division of the volume of the dispersed phase by the volume of the continuous phase. The standard deviation originated because of the unclear distribution of Span 80. However, we estimate the error of the determined phase volume to be lower than 10% relative.

#### Rheological characterization of selected emulsions

The major goal of these experiments was the confirmation of the existence of a yield point as is characteristic for Bingham fluids. The rheological behaviour of selected emulsions was determined with an AR 1000-N Rheometer (TA Instruments, New Castle, DE, USA) by using plate–plate geometry in shear stress controlled measurement mode. Plates with a diameter of 22 mm were applied and the gap size was 540 µm. The shear rate range was 1–60 s^−1^ and the measurement temperature was 25 °C for emulsions SA4 and SG4 and 40 °C for emulsions SA2 and SG2.

#### Tribological determination of adhesion and friction

The tribological measurements were performed with a nanotribometer NTR^2^ (CSM^©^ Instruments, Peseux, Switzerland) equipped with the dual beam cantilever STH-001. This cantilever features a highly sensitive dual beam spring, which is able to measure forces in the *x*- and *z*-direction with a resolution of 30 nN. Both adhesion and friction forces are detected by two independent high-resolution capacitive sensors, whereas a piezo actuator provides smooth and steady motion at a slow pace. The actual measuring head consists of an even and almost square-shaped SiO_2_-coated silicon wafer plate (SilChem, Freiberg, Germany) of 6.45 mm^2^ (4P02/50, orientation 100, bulk-doping with n/phosphorus). Its thickness amounted to 380 µm. Abrasive blast cleaning resulted in roughness values of 1.88 µm (Ra) and 2.40 µm (Rq), respectively. The surface energy of the pristine silicon wafer is 35 mN m^−1^ and 31 mN m^−1^ after abrasive blast cleaning, respectively. Before measurement, the solid emulsions were heated in a water bath to 35–40 °C yielding a spreadable consistency. The liquid emulsions were homogenized by the ultrasonic transducer UP200S at an intensity of 85% for two minutes. Afterwards, the emulsions were applied to a glass slide within an interspace (0.8 × 4.0 cm), confined by stripes of Sellotape^TM^ or Scotch^®^ Tape. The height of the tape was 43 μm leading to an emulsion film thickness of the highly viscous emulsions (WP20, WG20, VP50, VG50, SG2, OG2, SA4, SG4) of the same height. In the case of the aqueous, low viscous emulsions (SA2, OA2, SW2, OW2), 60 µL of one emulsion was applied onto the glass slide within the interspace and smoothed out with the tip of a pipette. Preliminary pretests showed that if we had used less than 60 µL, the liquid film within the interspace would have contracted (due to its high surface energy) from the outside towards the center, which would have prevented measurements on continuous and steady liquid films. The excess fluid was pressed out beyond the tape borders upon the displacement of the liquid due to the applied normal force. The measurements were performed at room temperature (≈22 °C).

#### Adhesion measurements

The adhesion force was determined by pressing a SiO_2_-coated silicon wafer plate on the emulsion film followed by continuously pulling it away perpendicularly to the surface. Once the wafer touched the surface with a contact load of 0.3 mN, the measurement started. The pressure was then increased up until 3.3 mN with a loading rate of 0.1 mN s^−1^. After being held at 3.3 mN for two seconds, the pressure decreased with an unloading rate of 0.1 mN s^−1^. At a load of 0.3 mN, the wafer was retracted with a speed of 33.3 μm s^−1^. The software Indentation 5.15 (CSM^©^ Instruments, Peseux, Switzerland) was used for recording and analysing the adhesion measurements. After the baseline had been set at a value before any pressure was applied, the lowest value represented the adhesion force.

In the experiments, the silicon wafer was immersed into a smear of the emulsion to be tested, pressed to the surface of the microscope slide and eventually retracted at a constant speed normal to the contact surface (without shear or pulling forces). In such a setup, which involves a relatively high amount of fluid, one mainly measures the adhesion that corresponds to the viscosity of the fluid as is typical in viscous (Stefan) adhesion regimes [65]. Our experiment is comparable with a probe tack test as commonly carried out for pressure sensitive adhesives (PSA). Wetting takes place as soon as the probe comes into contact with the emulsion. In our experiments, in most of the emulsions, failure was cohesive, since after separation, parts of the emulsion were present on both formed surfaces. In this case, the energy required for separation depends mainly on the viscosity of the emulsion and, in addition, on the formation of fibrils during the separation of the surfaces. Only in the four gelatin-containing emulsions (VG50, WG20, SG2, OG2) with solid consistence at room temperature did the wafer seem to separate directly at the surface of the emulsion, since no remnants of it were left on its surface after separation. This is indicative of the high viscosity and cross-linking of the adhesive leading to high cohesive strengths. The required force in this case depends mainly on the surface energies of the probe and the adhesive and to a minor extent to the deformation of the adhesive.

#### Friction measurements

The friction force was determined by dragging the SiO_2_-coated silicon wafer over the surface of the emulsion within the tape confined interspace with a load of 0.6 mN. The wafer was rubbed with an oscillating motion of 3–5 cycles over the surface, whereby one cycle represents one full forward and backward motion. Every emulsion was sampled at three different dragging speeds and distances, i.e., 50 μm s^−1^ (distance of 500 μm), 200 μm s^−1^ (distance of 700 μm) and 500 μm s^−1^ (distance of 800 μm). The friction curves were recorded and analysed with the software TriboX 4.4.T (CSM^©^ Instruments, Peseux, Switzerland). The last cycle was always ignored, because sometimes it was not completely finished. The friction force *F*_f_ was calculated via Equation 3:

[3]
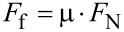


where μ is the friction coefficient and *F*_N_ is the normal load. For one measurement, the arithmetic mean of the sliding friction values of all performed cycles was calculated considering forward motion only.

#### Statistical analyses

In terms of the structural data, various descriptive statistics (e.g., mean value, median, excess kurtosis) were calculated from the combined frequency distributions of the droplet volumes measured by laser diffraction and by fluorescent and bright field images. Whereas volumes associated with droplet sizes >1.5 µm were taken from the microscopical analysis (Supporting Information File 1, Figure S3c), all volumes determined by fluorescent and bright field images belonging to droplets <1.5 μm were replaced by the results of the laser diffraction. As the limitation of the angular resolution of the microscope is approached, the reliability of droplet sizes <1.5 µm decreases, whereas the laser diffraction offers accurate resolution in the nanometer range.

With respect of the performance data (adhesion and friction), all the statistical analyses were performed on logarithmized values. Before logarithmization, the number one was added to all the values in order to avoid negative logarithms. Any statistical differences in adhesion and friction between the emulsions were tested by a nonparametric Kruskal–Wallis ANOVA followed by Kruskal–Wallis post hoc multiple comparisons. In addition, the grand means of the first and second generation of emulsions were compared with a Mann–Whitney U test. For a comparison of the influence of the various speeds in the friction experiments, a Friedman test followed Friedman post hoc multiple comparisons was performed.

Additionally, all descriptive, tribological and chemical parameters were correlated against each other to reveal any relationships between these categories. For this analysis, the structural parameters of the two controls water and squalane were defined by 0. Moreover, deviating from our descriptive statistics (Supporting Information File 1, Table S1), the phase volume ratio was calculated by dividing the hydrophobic (oily) phase by the hydrophilic (watery) phase. This was necessary to attain a consistent interpretation of the results with respect to o/w and w/o emulsions. All the statistical analyses were performed with IBM^®^ SPSS^®^ Statistics 22 on logarithmized values. Before logarithmization, the number one was added to all the values of the chemical and tribological parameters and the descriptive parameters were increased by 2.573 to avoid negative logarithms (the excess kurtosis −1.573 of the emulsion WG20 was the lowest value).

The levels of significance were classified into four groups symbolized by asterisks (*), i.e., * representing *p* < 0.05, ** representing *p* < 0.01 and * * * representing *p* < 0.001. One asterisk in brackets (*) represents an almost significant test result at the 10% significance level, indicating that there is at least a trend towards significance.

## Supporting Information

Supporting Information features additional emulsion images of both the first and the second generation, structural characteristics of all the emulsions, values of the adhesion and friction experiments and the specific test statistics.

File 1 Additional figures and tables.
