# Supplementary material for: Structural and tribometric characterization of biomimetically inspired synthetic "insect adhesives"
Source: Beilstein J Nanotechnol. 2017 Jan 6;8:45–63. doi: 10.3762/bjnano.8.6 (PMC5238622; doi:10.3762/bjnano.8.6)
Supplement: File 1 — Additional figures and tables. [file Beilstein_J_Nanotechnol-08-45-s001.pdf]

Supporting Information

for

Structural and tribometric characterization of biomimetically inspired synthetic "insect adhesives"

Matthias W. Speidel[1], Malte Kleemeier[2], Andreas Hartwig[2,3], Klaus Rischka[2], Angelika Ellermann[4], Rolf Daniels[4] and Oliver Betz[1*]

Address: [1]Institut für Evolution und Ökologie, Universität Tübingen, Auf der Morgenstelle 28, D-72076 Tübingen, Germany, [2]Fraunhofer-Institut für Fertigungstechnik und Angewandte Materialforschung, Wiener Str. 12, D-28359 Bremen, Germany, [3]Universität Bremen, Fachbereich 2 Biologie/Chemie, Leobener Str., 28359 Bremen, Germany and [4]Pharmazeutisches Institut, Universität Tübingen, Pharmazeutische Technologie und Biopharmazie, Auf der Morgenstelle 8, D-72076 Tübingen, Germany

Email: Oliver Betz - oliver.betz@uni-tuebingen.de

[*] Corresponding author

Additional figures and tables

1. Figures

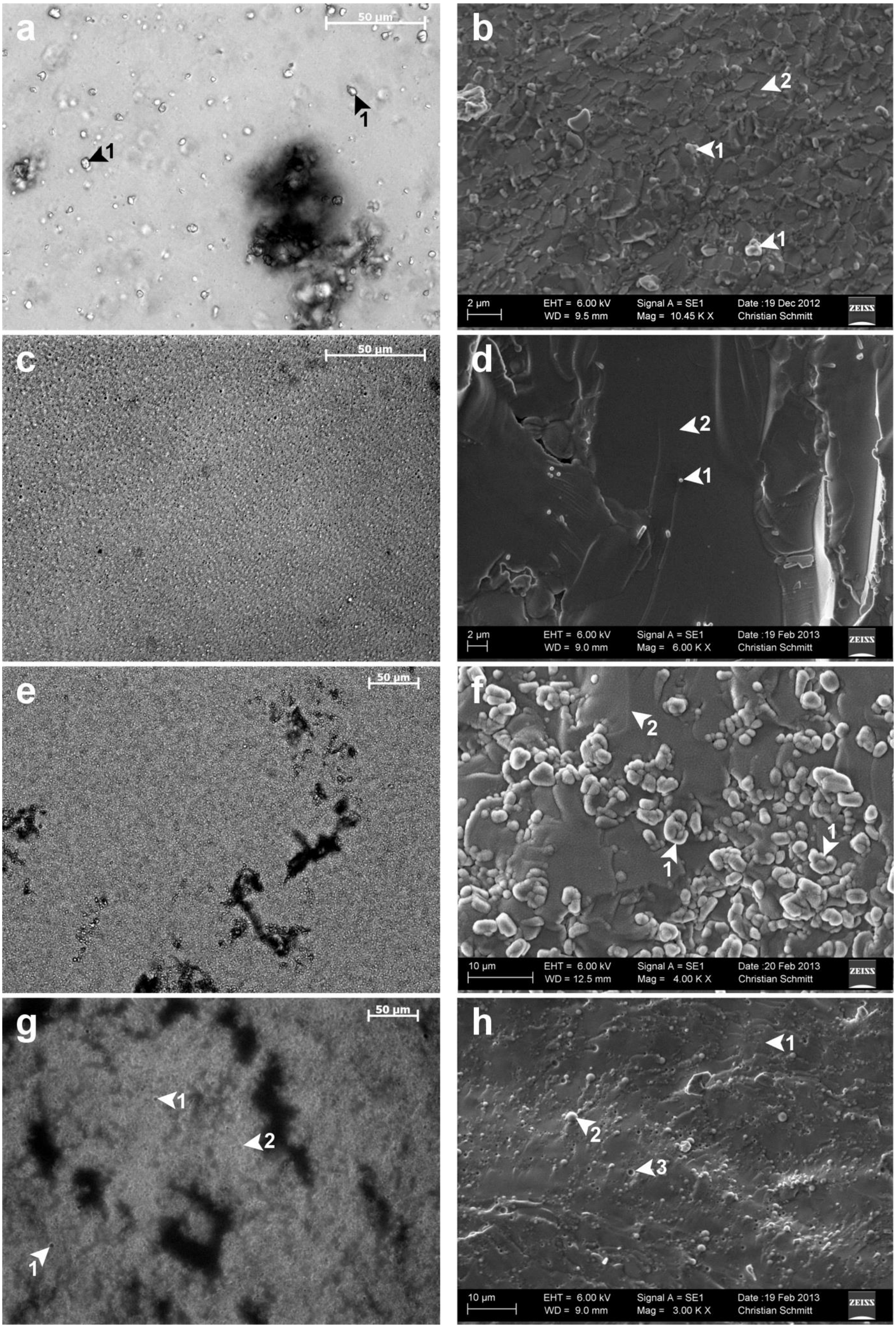
a
50 µm
1
1
b
2
1
1
2 µm
EHT = 6.00 kV
WD = 9.5 mm
Signal A = SE1
Mag = 10.45 K X
Date :19 Dec 2012
Christian Schmitt
ZEISS
c
50 µm
d
2
1
2 µm
EHT = 6.00 kV
WD = 9.0 mm
Signal A = SE1
Mag = 6.00 K X
Date :19 Feb 2013
Christian Schmitt
ZEISS
e
50 µm
f
2
1
1
10 µm
EHT = 6.00 kV
WD = 12.5 mm
Signal A = SE1
Mag = 4.00 K X
Date :20 Feb 2013
Christian Schmitt
ZEISS
g
50 µm
1
2
1
h
1
2
3
10 µm
EHT = 6.00 kV
WD = 9.0 mm
Signal A = SE1
Mag = 3.00 K X
Date :19 Feb 2013
Christian Schmitt
ZEISS

Figure S1: Droplet distribution of selected emulsions of the first and second generation. Left side: appearance of the emulsions by bright field light or fluorescence microscopy; right side: appearance of the emulsions by cryo-SEM. (a) Loosely scattered and polyhedral droplets (1) of the emulsion OG2 by light microscopy. The emulsion was examined after dilution with water under bright field. The lipid phase octacosane consisted of solid polyhedral fragments causing aggregation. (b) Polyhedral fragments (1) of the emulsion OG2 embedded in outer phase (2) of flocculent structure. Main size range of fragments about 1 - 2 µm. (c) Extremely dense distribution of tiny droplets of the protein-free emulsion SW2 (mainly black appearance) by light microscopy. Many droplets below microscopic resolution. (d) Widely scattered droplets (1) of the emulsion SW2 embedded in the outer phase (2) of smooth appearance. Main size range of droplets about <1 µm. (e) Extremely dense distribution of tiny droplets of the protein-free emulsion OW2 by light microscopy. Many droplets below microscopic resolution. The emulsion was examined after dilution with water under bright field. (f) Aggregated polyhedral fragments (1) of emulsion OW2 embedded in a smooth outer phase (2). Main size range of fragments about <1 - 5 µm. (g) Non-fluorescent droplets (1) of emulsion SG4 embedded in fluorescent outer phase (2) (the Sudan-III-stained emulsion was examined under light excitation of 530 – 560 nm) after dilution with squalane. The non-fluorescent droplets in combination with the fluorescent outer phase are indicative of a w/o emulsion. (h) Smooth emulsion surface (1) of SG4 consisting of both droplets (2) and cavities (3). The cavities (<1 - 2 µm) seem to be remains of water droplets that evaporated during the sublimation step, whereas the unaffected droplets probably represent droplets that have not fully evaporated. Main size range of visible droplets about <1 - 2 µm.

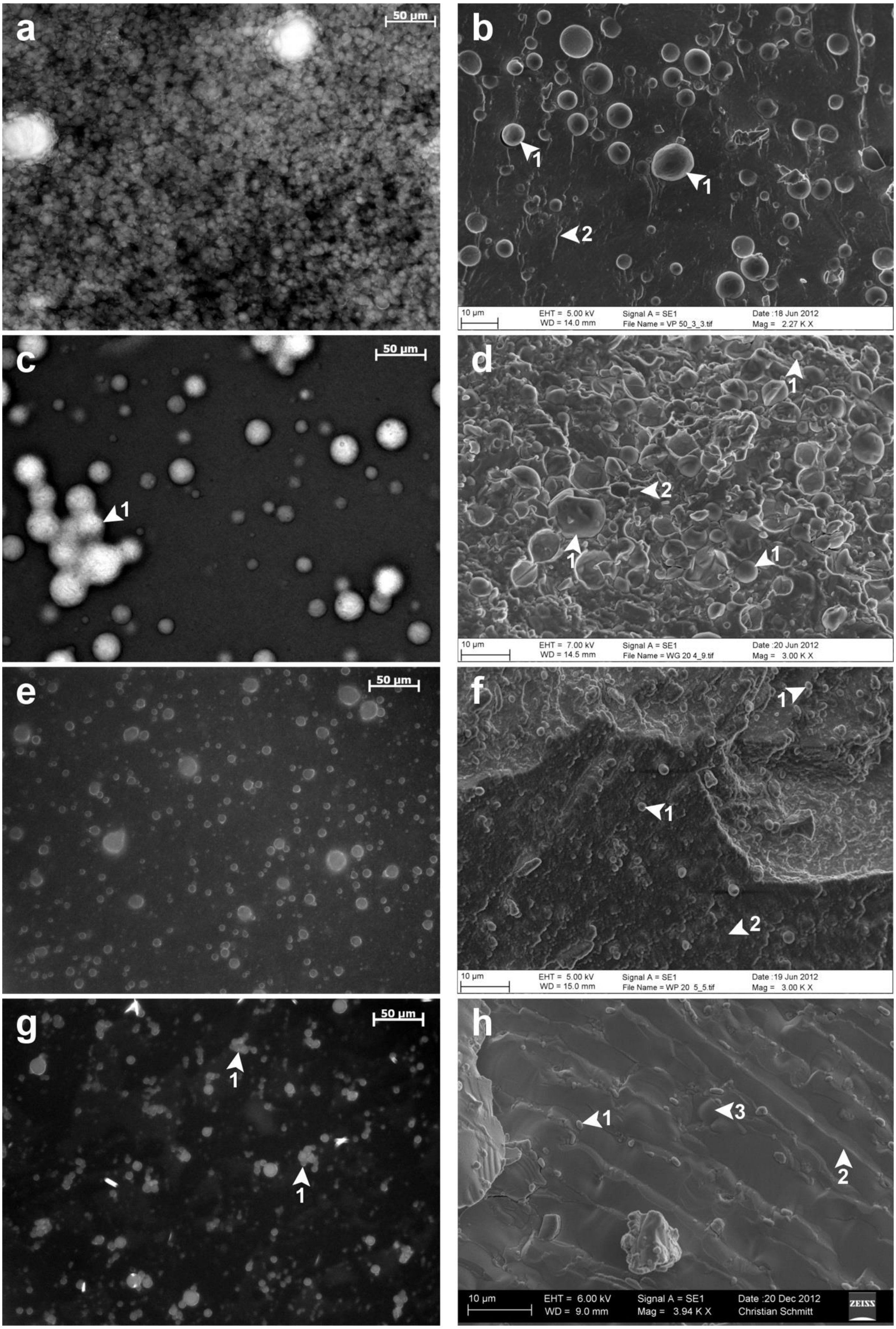

a
50 µm
b
1
1
2
10 µm
EHT = 5.00 kV
WD = 14.0 mm
Signal A = SE1
File Name = VP 50_3_3.tif
Date :18 Jun 2012
Mag = 2.27 K X
c
50 µm
1
d
1
2
1
1
10 µm
EHT = 7.00 kV
WD = 14.5 mm
Signal A = SE1
File Name = WG 20 4_9.tif
Date :20 Jun 2012
Mag = 3.00 K X
e
50 µm
f
1
1
2
10 µm
EHT = 5.00 kV
WD = 15.0 mm
Signal A = SE1
File Name = WP 20_5_5.tif
Date :19 Jun 2012
Mag = 3.00 K X
g
50 µm
1
1
h
1
3
2
10 µm
EHT = 6.00 kV
WD = 9.0 mm
Signal A = SE1
Mag = 3.94 K X
Date :20 Dec 2012
Christian Schmitt

Figure S2: Droplet distribution of selected emulsions of the first and second generation. Left side: appearance of the emulsions by bright field light or fluorescence microscopy; right side: appearance of the emulsions by cryo-SEM. (a) Densely distributed fluorescent lipid droplets of emulsion VP50 (Sudan-III-stained emulsion was examined under light excitation of 300 - 400 nm). The fluorescent droplets are indicative of the o/w characteristic of this emulsion, because these fluorescent oily droplets form the inner phase. The outer hydrophilic phase is reduced because of evaporation. (b) Homogeneously distributed droplets (arrow 1) of emulsion VP50 embedded in an outer phase (2) with a slightly rough appearance. The emulsion contained a droplet size in the range of about <1 - 20 µm, but the size of most of the droplets lies between 2 - 10 µm. (c) Loosely scattered droplets of the emulsion WG20 by light microscopy. Sporadic aggregation of droplets to larger fragments (1). (d) Distributed droplets (1) of emulsion WG20 embedded in an outer phase (2) with a rough and chiselled appearance. Main size range of irregularly shaped droplets about 4 - 10 µm. (e) Homogeneously distributed fluorescent lipid droplets of emulsion WP20 (Sudan-III-stained emulsion was examined under light excitation of 530 – 560 nm) after dilution with water. The fluorescent droplets are indicative of the o/w characteristic of the emulsion WP20, because these fluorescent oily droplets form the inner phase. (f) Densely distributed droplets (1) of emulsion WP20 embedded in an outer phase (2) of a rough appearance. Main size range of round- to oval-shaped droplets about 1 - 10 µm. (g) Fluorescent droplets of emulsion SA2 embedded in a non-fluorescent outer phase (the Sudan-III-stained emulsion was examined under light excitation of 530 – 560 nm). The fluorescent droplets in combination with the non-fluorescent outer phase are indicative of a o/w emulsion. Frequent occurrence of aggregated droplets (1). (h) Widely scattered droplets (1) of emulsion SA2 embedded in the outer phase (2) of a smooth appearance and a lamellar-like structure. Round bulges (3) of the surface might indicate hidden large droplets under a layer of the outer phase. Main size range of droplets about <1 - 2 µm.

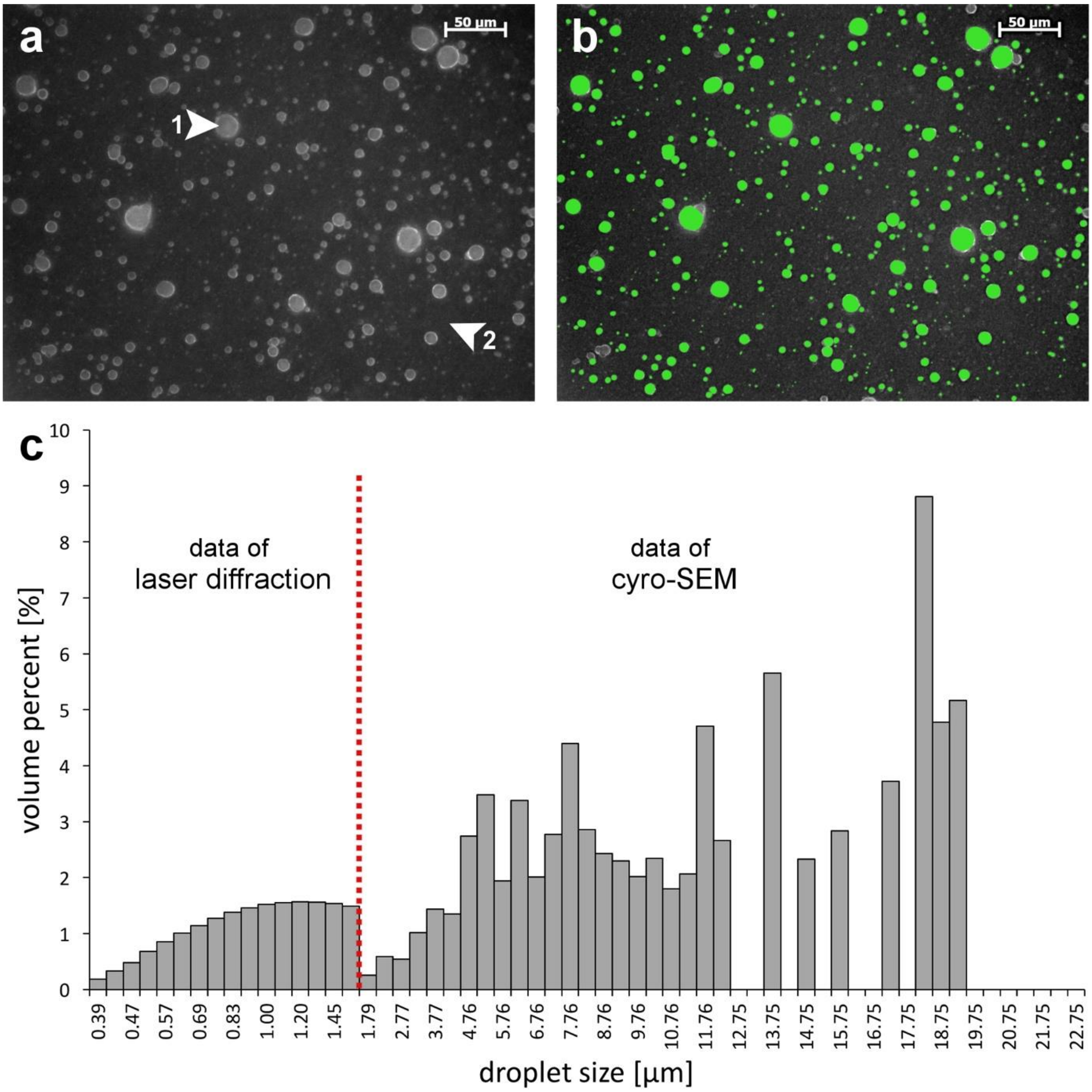

Figure S3: Exemplified droplet-size distribution of the first generation emulsion WP20 (o/w) as established by microscopical analysis. (a) Raw fluorescence image. The lipid phase was amplified by Sudan III to maintain fluorescence. Arrow 1 shows fluorescent lipophilic droplets, whereas arrow 2 shows the non-fluorescent hydrophilic phase. (b) Further preparation of fluorescence image for the droplet-size distribution analysis. All clearly visible droplets were coloured in an intense green by using Adobe® Photoshop® CS4 11.0. (c) Histogram showing the volume percentage of the various droplet sizes. Droplet-sizes of > 1.5 µm were semi-automatically determined by the image processing software ImageJ (W. Rasband, version 1.47n), whereas droplet sizes of 0 - 1.5 µm were identified by laser diffraction. The droplet sizes were transformed into volume percentage by equation (2). Emulsion WP20 is characterized by a wide droplet-size distribution ranging from extremely small to large droplets.

2. Tables

Table S1: Detailed emulsion characteristics as determined by direct evaluation and experiments at room temperature (22 °C) or indirectly calculated from the deployed oil and water phase. The main size range of droplets as established by light / fluorescence microscopy was determined within the range of the 15% and 85% quantiles. The droplets sizes determined by cryo-SEM were determined by single measurements of about 20 randomly distributed droplets. The phase volume ratio was calculated by the equation: $phase\ volume\ ratio = \frac{V_d}{V_c}$, where V_d is the volume of the dispersed phase and V_c is the volume of the continuous phase. Only if the skewness was > 1.0 (or < -1.0) was it considered substantial. For excess kurtosis, a z-score was obtained by dividing the excess kurtosis by its standard error. For z-scores > 1.96 (or < -1.96), the excess kurtosis was considered substantial. Otherwise, the droplet distribution was classified as almost mesokurtic.

**emulsion**	**generation**	**general appearance**	**light / fluorescence microscopy**	**cryo-SEM**	**descriptive parameters (determined by light / fluorescence microscopy)**	**phase volume ratio (directly calculated from the deployed oil and water phase)**
VG50<br>(Fig. 1 a, b)	1	solid; rubber-like;<br>not sticky	homogeneous and dense distribution of droplets; main size range about 5.5 - 16.3 µm; individual droplets sizes > 100 µm; o/w emulsion	very dense homogeneous distribution of droplets; surface of outer phase of slightly rough appearance; main size range of droplets about 5 - 15 µm; individual droplet sizes range between < 1 µm and ≤ 50 µm; smooth droplets surrounded by a slightly textured boundary layer	10 % percentile = 1.00 µm<br>25 % percentile = 12.21 µm<br>75 % percentile = 65.50 µm<br>90 % percentile = 102.00 µm<br>median = 22.00 µm<br>arithmetic mean = 37.68 µm<br>mode = 102.00 µm<br>standard deviation = 36.44<br>excess kurtosis = -0.70 (almost mesokurtic)<br>skewness = 0.91 (almost symmetrical)	oil phase volume = 30.00 ml<br>water phase volume = 29.58 ml<br>phase volume ratio = 1.014
VP50<br>(Supporting Information: Fig. S2 a, b)	1	soft; elastic;<br>slightly sticky	homogeneous and dense distribution of droplets; main size range about 4.4 - 12.2 µm; individual droplet sizes ≤ 27 µm; o/w emulsion	loose homogeneous distribution of droplets; surface of outer phase of slightly rough appearance; main size range of droplets about 2 - 10 µm; individual droplet sizes range between < 1µm and ≤ 20µm; smooth droplets	10 % percentile = 1.00 µm<br>25 % percentile = 5.39 µm<br>75 % percentile = 18.25 µm<br>90 % percentile = 24.09 µm<br>median = 12.60 µm<br>arithmetic mean = 12.31 µm<br>mode = 18.25 µm<br>standard deviation = 8.26<br>excess kurtosis = -1.11 (platykurtic)<br>skewness = 0.06 (almost symmetrical)	oil phase volume = 30.00 ml<br>water phase volume = 30.00 ml<br>phase volume ratio = 1.00

emulsion	generation	general appearance	light / fluorescence microscopy	cryo-SEM	descriptive parameters (determined by light / fluorescence microscopy)	phase volume ratio (directly calculated from the deployed oil and water phase)
WG20 (Supporting Information: Fig. S2 c, d)	1	solid;<br>under pressure brittle;<br>not sticky	homogeneous and loose distribution of droplets; main size range about 6.8 - 24.8 µm; individual droplet size range < 1 µm (below microscopic resolution) and ≤ 40 µm; sporadic aggregation of droplets to larger fragments; o/w emulsion	dense homogeneous distribution of droplets; surface of outer phase of rough and chiselled appearance; main size range of droplets about 4 - 10 µm; individual droplets < 1 µm in between larger droplets; droplets about 50 µm were rare; droplets irregularly shaped	10 % percentile = 0.47 µm<br>25 % percentile = 0.57 µm<br>75 % percentile = 26.62 µm<br>90 % percentile = 32.59 µm<br>median = 11.93 µm<br>arithmetic mean = 13.62 µm<br>mode = 0.52 µm<br>standard deviation = 13.64<br>excess kurtosis = -1.57 (platykurtic)<br>skewness = 0.30 (almost symmetrical)	oil phase volume = 12.00 ml<br>water phase volume = 47.32 ml<br>phase volume ratio = 0.254
WP20 (Supporting Information: Fig. S2 e, f)	1	solid; rubber-like;<br>under pressure brittle;<br>not sticky	homogeneous and dense distribution of droplets; main size range about 1.6 – 6.3 µm; individual droplet size range between < 1 µm (below microscopic resolution) and ≤ 40 µm; o/w emulsion	dense distribution of droplets; surface of outer phase of rough appearance; main size range of droplets about 1 – 10 µm; individual droplet sizes range between < 1 µm and about 50 µm; droplets round to oval-shaped	10 % percentile =1.00 µm<br>25 % percentile = 4.76 µm<br>75 % percentile = 15.73 µm<br>90 % percentile = 18.65 µm<br>median = 9.26 µm<br>arithmetic mean = 9.75 µm<br>mode = 18.25 µm<br>standard deviation = 6.23<br>excess kurtosis = -1.26 (platykurtic)<br>skewness = 0.07 (almost symmetrical)	oil phase volume = 12.00 ml<br>water phase volume = 48.00 ml<br>phase volume ratio = 0.25

**emulsion**	**generation**	**general appearance**	**light / fluorescence microscopy**	**cryo-SEM**	**descriptive parameters (determined by light / fluorescence microscopy)**	**phase volume ratio (directly calculated from the deployed oil and water phase)**
SA2<br>(Supporting Information: Fig. S2 g, h)	2	watery consistency;<br>unstable phases;<br>creaming of oily phase;<br>not sticky	homogeneous and loose distribution of droplets; main size range about 2.7 - 8.0 µm; individual droplet size range between < 1 µm (below microscopic resolution) and ≤ 30 µm; sporadic aggregation of droplets to larger fragments; fluorescent droplets and successful dilution of outer phase with water indicative of o/w emulsion	widely scattered droplets; surface of outer phase of smooth appearance showing a lamellar structure; main size range of droplets about < 1 - 2 µm; round bulges on surface might be indicative of hidden larger droplets of about 5 µm, droplets round to oval-shaped	10 % percentile = 1.41 µm<br>25 % percentile = 6.54 µm<br>75 % percentile = 28.51 µm<br>90 % percentile = 31.51 µm<br>median = 9.53 µm<br>arithmetic mean = 14.65 µm<br>mode = 31.51 µm<br>standard deviation = 11.02<br>excess kurtosis = -1.34 (platykurtic)<br>skewness = 0.50 (almost symmetrical)	oil phase volume = 3.00 ml<br>water phase volume = 49.47 ml<br>phase volume ratio = 0.061 ± 0.012[1]
SG2<br>(Fig. 1 c, d)	2	solid; rubber-like;<br>under pressure brittle;<br>liquefaction by warming to 40 - 45 °C;<br>not sticky	homogeneous and dense distribution of droplets; main size range about 2.6 - 4.5 µm; individual droplet size < 1 µm (below microscopical resolution); fluorescent droplets and successful dilution of outer phase with water are indicative of o/w emulsion	homogeneously distributed droplets; surface of outer phase of very rough appearance with flaked structure, main size range of droplets about < 1 - 5 µm; droplets round to oval-shaped	10 % percentile = 2.77 µm<br>25 % percentile = 3.45 µm<br>75 % percentile = 5.15 µm<br>90 % percentile = 5.76 µm<br>median = 4.26 µm<br>arithmetic mean = 4.26 µm<br>mode = 3.77 µm<br>standard deviation = 1.11<br>excess kurtosis = 0.32 (almost mesokurtic)<br>skewness = 0.02 (almost symmetrical)	oil phase volume = 3.00 ml<br>water phase volume = 49.47 ml<br>phase volume ratio = 0.061 ± 0.012[1]

emulsion	generation	general appearance	light / fluorescence microscopy	cryo-SEM	descriptive parameters (determined by light / fluorescence microscopy)	phase volume ratio (directly calculated from the deployed oil and water phase)
OA2<br>(Fig. 1 e, f)	2	watery consistency;<br>unstable phases;<br>formation of clumps;<br>not sticky	homogeneous and loose distribution of droplets; main size range about 2.1 - 4.0 µm; individual droplet sizes < 1 µm (below microscopic resolution); sporadic aggregation of droplets to larger fragments; droplets polyhedral; fluorescent droplets and successful dilution of outer phase with water are indicative of o/w emulsion	widely scattered droplets; surface of outer phase of smooth appearance showing a lamellar structure; main size range of droplets about < 1 - 3 µm; droplets polyhedral	10 % percentile = 1.48 µm<br>25 % percentile = 3.27 µm<br>75 % percentile = 4.26 µm<br>90 % percentile = 4.26 µm<br>median = 3.77 µm<br>arithmetic mean = 3.48 µm<br>mode = 4.26 µm<br>standard deviation = 1.11<br>excess kurtosis = 0.79 (almost mesokurtic)<br>skewness = -1.12 (neg. skewness)	oil phase volume = 2.54 ml<br>water phase volume = 49.47 ml<br>phase volume ratio = 0.051 ± 0.012[1]
OG2<br>(Supporting Information: Fig. S1 a, b)	2	solid;<br>rubber-like;<br>under pressure brittle;<br>liquefaction by warming to 40 - 45 °C;<br>not sticky	homogeneous and loose distribution of droplets; main size range about 1.9 - 3.9 µm; individual droplet sizes < 1 µm (below microscopic resolution); sporadic aggregation of droplets to large flakes; droplets polyhedral; fluorescent droplets and successful dilution of outer phase with water are indicative of o/w emulsion	dense distribution of droplets; outer phase flocculent; main size range of droplets about 1 - 2 µm; single droplets about 6 µm; droplets polyhedral	10 % percentile = 1.60 µm<br>25 % percentile = 2.77 µm<br>75 % percentile = 4.76 µm<br>90 % percentile = 5.26 µm<br>median = 3.77 µm<br>arithmetic mean = 3.58 µm<br>mode = 4.76 µm<br>standard deviation = 1.27<br>excess kurtosis = -0.31 (almost mesokurtic)<br>skewness = -0.48 (almost symmetrical)	oil phase volume = 1.98 ml<br>water phase volume = 49.18 ml<br>phase volume ratio = 0.051 ± 0.012[1]

**emulsion**	**generation**	**general appearance**	**light / fluorescence microscopy**	**cryo-SEM**	**descriptive parameters (determined by light / fluorescence microscopy)**	**phase volume ratio (directly calculated from the deployed oil and water phase)**
SW2<br>(Supporting Information: Fig. S1 c, d)	2	watery consistency;<br>not sticky	very dense distribution of tiny droplets; many droplets < 1 µm (below microscopic resolution); main size range about < 1 - 1.2 µm; fluorescent droplets and successful dilution of outer phase with water are indicative of o/w emulsion	widely scattered droplets; surface of outer phase of smooth appearance; main size range of droplets about ≤ 1 µm	10 % percentile = 0.39 µm<br>25 % percentile = 0.43 µm<br>75 % percentile = 0.52 µm<br>90 % percentile = 1.79 µm<br>median = 0.47 µm<br>arithmetic mean = 0.77 µm<br>mode = 0.43 µm<br>standard deviation = 0.62<br>excess kurtosis = 0.36 (almost mesokurtic)<br>skewness = 1.46 (pos. skewness)	oil phase volume = 3.00 ml<br>water phase volume = 46.29 ml<br>phase volume ratio = 0.065 ± 0.013[1]
OW2<br>(Supporting Information: Fig. S1 e, f)	2	watery consistency;<br>unstable phases;<br>formation of clumps;<br>not sticky	very dense distribution of tiny droplets; many droplets < 1 µm (below microscopic resolution); main size range about ≤ 1 µm, many flakes of aggregated droplets; fluorescent droplets and successful dilution of outer phase with water are indicative of o/w emulsion	droplets aggregated and mainly localized in scattered areas; surface of outer phase of smooth appearance; main size range of droplets about < 1 - 5 µm; droplets polyhedral	10 % percentile = 0.41 µm<br>25 % percentile = 0.50 µm<br>75 % percentile = 0.72 µm<br>90 % percentile = 0.86 µm<br>median = 0.60 µm<br>arithmetic mean = 0.63 µm<br>mode = 0.60 µm<br>standard deviation = 0.22<br>excess kurtosis = 14.77 (leptokurtic)<br>skewness = 3.11 (pos. skewness)	oil phase volume = 2.53 ml<br>water phase volume = 46.29 ml<br>phase volume ratio = 0.055 ± 0.013[1]

emulsion	generation	general appearance	light / fluorescence microscopy	cryo-SEM	descriptive parameters (determined by light / fluorescence microscopy)	phase volume ratio (directly calculated from the deployed oil and water phase)
SA4 (Fig. 1 g, h)	2	oily consistency; not sticky	droplets embedded in dense outer phase; main size range about 2.3 - 4.7 µm; no fluorescent droplets, but fluorescent outer phase; dilution with squalane impossible, but dilution with water possible; presumably w/o emulsion	homogeneous distribution of droplets; surface of outer phase of smooth appearance; main size range of droplets about 1 - 4 µm; outer phase carpeted with round cavities of size range of < 1 – 2 µm; droplets round	10 % percentile = 2.77 µm<br>25 % percentile = 3.77 µm<br>75 % percentile = 5.76 µm<br>90 % percentile = 6.85 µm<br>median = 4.76 µm<br>arithmetic mean = 4.74 µm<br>mode = 4.76 µm<br>standard deviation = 1.77<br>excess kurtosis = 1.63 (leptokurtic)<br>skewness = 0.77 (almost symmetrical)	oil phase volume = 38.77 ml<br>water phase volume = 19.34 ml<br>phase volume ratio = 0.50 ± 0.34[1]
SG4 (Supporting Information: Fig. S1 g, h)	2	oily consistency; not sticky	droplets embedded in dense outer phase; main size range about 2.3 - 3.5 µm; no fluorescent droplets, but fluorescent outer phase; dilution with both squalane and water impossible; presumably w/o emulsion	homogeneous distribution of droplets; surface of outer phase of smooth appearance; main size range of droplets about < 1 - 2 µm; outer phase carpeted with round cavities of size range of < 1 - 2 µm; droplets round	10 % percentile = 2.28 µm<br>25 % percentile = 2.77 µm<br>75 % percentile = 3.77 µm<br>90 % percentile = 5.76 µm<br>median = 3.27 µm<br>arithmetic mean = 3.57 µm<br>mode = 2.77 µm<br>standard deviation = 1.03<br>excess kurtosis = 0.21 (almost mesokurtic)<br>skewness = 1.00 (pos. skewness)	oil phase volume = 38.77 ml<br>water phase volume = 19.34 ml<br>phase volume ratio = 0.50 ± 0.34[1]

[1]The standard deviation of the phase volume ratio represents the three possible distributions of the non-ionic surfactant Span 80: (1) Span 80 fully dissolves in the hydrophilic fraction, (2) Span 80 fully dissolves in the hydrophobic fraction or (3) the fatty acid section of Span 80 dissolves in the hydrophobic fraction, whereas the sorbitan section dissolves in the hydrophilic fraction. With regard to the structure of the non-ionic surfactant Span 80, about 66% of the molecule consists of the fatty acid oleat (Supporting Information: Tab. S16), which can be credited to the hydrophobic phase, whereas the remaining 34% belongs to the hydrophilic phase.

Table S2: Arithmetic means ± standard deviations of both the adhesion and the friction values for each emulsion as determined in the tribological measurements. The measured values refer to the surface area of the silicon wafer of 6.45 mm^2. N describes the number of measurements of one emulsion. After each measurement, the applied emulsion was renewed.

**emulsion**	**adhesion [mN]**	**friction [mN]**		
		**50 µm/s**	**200 µm/s**	**500 µm/s**
VG50	31.02 ±15.6<br>N=10	21.60 ±10.4<br>N=11	39.17 ±11.6<br>N=11	32.12 ±10.3<br>N=10
VP50	93.41±37.6<br>N=9	5.25 ±2.8<br>N =10	13.93 ±8.0<br>N =16	19.37 ±6.5<br>N =10
WG20	51.18 ±17.9<br>N=10	39.14 ±8.9<br>N=9	28.90 ±6.9<br>N=11	18.07 ±3.7<br>N=10
WP20	57.12 ±27.6<br>N=12	3.73 ±1.1<br>N=10	11.36 ±7.5<br>N=10	20.25 ±4.7<br>N=10
SA2	0.93 ±0.8<br>N=9	0.27 ±0.1<br>N=10	0.38 ±0.2<br>N=9	0.40 ±0.1<br>N=8
SG2	15.49 ±9.5<br>N=10	4.00 ±1.3<br>N=7	2.68 ±0.3<br>N=7	5.71 ±1.3<br>N=7
OA2	1.06 ±1.0<br>N=6	0.17 ±0.1<br>N=7	0.15 ±0.1<br>N=10	0.17 ±0.1<br>N=10
OG2	12.90 ±8.1<br>N=10	1.76 ±1.1<br>N=9	1.03 ±0.5<br>N=10	1.00 ±0.8<br>N=9
SW2	1.34 ±1.4<br>N=10	0.13 ±0.1<br>N=10	0.22 ±0.1<br>N=9	0.21±0.2<br>N=7
OW2	1.03 ±1.0<br>N=10	0.07 ±0.0<br>N=8	0.07 ±0.0<br>N=8	0.08 ±0.0<br>N=8
SA4	17.63 ±8.8<br>N=10	0.21 ±0.2<br>N=8	0.42 ±0.3<br>N=9	0.46 ±0.4<br>N=9
SG4	15.42 ±5.3<br>N=10	0.20±0.1<br>N=10	0.33 ±0.1<br>N=10	0.32 ±0.1<br>N=10
water	1.26 ±0.8<br>N=9	0.14 ±0.1<br>N=9	0.24 ±0.1<br>N=9	0.24 ±0.1<br>N=8
squalane	3.04 ±2.1<br>N=10	0.61 ±0.4<br>N=11	1.16 ±0.5<br>N=10	1.19 ±0.6<br>N=10
glass	0	0.16 ±0.2<br>N=5	0.08 ±0.0<br>N=6	0.12 ±0.1<br>N=5

Table S3: Listing of all significant pairwise comparisons of the logarithmized adhesion of the emulsions of the first generation. The results of pairwise comparisons that are not listed are not significant. Statistical analyses: Kruskal-Wallis ANOVA followed by Kruskal-Wallis post hoc multiple comparisons.

compared emulsions	test statistics	standardized test statistics	statistical significance
water – WG20	24.4	3.6	***
water – WP20	25.7	4.0	***
water – VP50	36.2	5.3	***
VG50 – VP50	-21.5	-3.2	**
water – VG50	14.7	2.2	*

Table S4: Listing of all significant pairwise comparisons of the logarithmized adhesion of the emulsions of the second generation. The results of all pairwise comparisons that are not listed are not significant. Statistical analyses: Kruskal-Wallis ANOVA followed by Kruskal-Wallis post hoc multiple comparisons.

compared emulsions	test statistics	standardized test statistics	statistical significance
SA2-OG2	-46.2	-3.6	***
SA2-SG2	-49.4	-3.9	***
SA2-SG4	-51.5	-4.0	***
SA2-SA4	-53.4	-4.2	***
OW2-OG2	44.2	3.7	***
OW2-SG2	47.4	3.9	***
OW2-SG4	-49.5	-4.1	***
OW2-SA4	-51.4	-4.3	***
OA2-SA4	-49.4	-3.6	***
water-SG2	43.5	3.5	***
water-SG4	45.6	3.7	***
water-SA4	47.5	3.8	***
OA2-SG2	45.4	3.3	**
OA2-SG4	-47.5	-3.4	**
water-OG2	40.3	3.3	**
OA2-OG2	-42.2	-3.0	**
squalane-SA4	34.7	2.9	**
squalane-SG4	32.8	2.7	**
squalane-SG2	30.7	2.5	*
squalane-OG2	27.5	2.3	*

Table S5: Listing of all significant pairwise comparisons of the the logarithmized friction between the emulsions of the first generation at the speed of 50 µm s^{-1}. The results of all pairwise comparisons that are not listed are not significant. Statistical analyses: Kruskal-Wallis ANOVA followed by Kruskal-Wallis post hoc multiple comparisons.

compared emulsions	test statistics	standardized test statistics	statistical significance
glass-VG50	34.8	4.0	***
glass-WG20	43.3	5.0	***
water-VG50	32.5	4.5	***
water-WG20	41.0	5.6	***
WP20-WG20	26.0	3.6	***
VP50-WG20	-23.3	-3.2	**
WP20-VG50	17.5	2.5	*
water-VP50	17.7	2.4	*
glass-VP50	20.0	2.3	*
VP50-VG50	14.8	2.1	*
water-WP20	15.0	2.0	*
glass-WP20	17.3	2.0	*

Table S6: Listing of all significant pairwise comparisons of the logarithmized friction between the emulsions of the first generation at the speed of 200 µm s^{-1}. The results of all pairwise comparisons that are not listed are not significant Statistical analyses: Kruskal-Wallis ANOVA followed by Kruskal-Wallis post hoc multiple comparisons.

compared emulsions	test statistics	standardized test statistics	statistical significance
glass-WG20	42.6	4.6	***
glass-VG50	49.2	5.5	***
water-WG20	35.3	4.2	***
water-VG50	42.0	5.3	***
glass-VP50	27.7	3.3	**
WP20-VG50	25.7	3.3	**
VP50-VG50	21.6	3.1	**
water-VP50	20.4	2.8	**
glass-WP20	23.5	2.6	*
WP20-WG20	19.0	2.3	*
VP50-WG20	-14.9	-2.0	*
water-WP20	16.3	2.0	*

Table S7: Listing of all significant pairwise comparisons of the logarithmized friction between the emulsions of the first generation at the speed of 500 µm s^{-1}. The results of all pairwise comparisons that are not listed are not significant. Statistical analyses: Kruskal-Wallis ANOVA followed by Kruskal-Wallis post hoc multiple comparisons.

compared emulsions	test statistics	standardized test statistics	statistical significance
glass-VG50	41.4	4.8	***
water-VG50	36.5	4.9	***
glass-VP50	27.6	3.2	**
glass-WP20	27.9	3.2	**
water-VP50	22.7	3.1	**
water-WP20	23.0	3.1	**
glass-WG20	23.7	2.8	**
water-WG20	18.9	2.6	*
WG20-VG50	17.7	2.6	*
VP50-VG50	13.8	2.0	*

Table S8: Listing of all significant pairwise comparisons of the logarithmized friction between the emulsions of the second generation at the speed of 50 µm s^{-1}. The results of all pairwise comparisons that are not listed are not significant. Statistical analyses: Kruskal-Wallis ANOVA followed by Kruskal-Wallis post hoc multiple comparisons.

compared emulsions	test statistics	standardized test statistics	statistical significance
OW2-squalane	-51.9	-4.1	***
OW2-OG2	-70.2	-5.2	***
OW2-SG2	77.2	5.4	***
glass-OG2	59.9	4.1	***
glass-SG2	67.0	4.4	***
SW2-OG2	-54.1	-4.3	***
SW2-SG2	-61.2	-4.5	***
water-OG2	51.9	4.0	***
water-SG2	59.0	4.2	***
OA2-OG2	-51.1	-3.7	***
OA2-SG2	58.1	4.0	***
OW2-SA2	45.2	3.5	**
SA4-SG2	48.1	3.4	**
SG4-SG2	45.5	3.4	**
SA4-OG2	41.0	3.1	**
SG4-OG2	38.4	3.0	**
glass-squalane	41.7	3.0	**
SW2-squalane	-35.9	-3.0	**
water-squalane	-33.7	-2.7	**
glass-SA2	35.0	2.5	*
OA2-squalane	-32.8	-2.5	*
OW2-SG4	-31.8	-2.4	*
SW2-SA2	-29.1	-2.4	*
SA2-SG2	-32.1	-2.4	*
water-SA2	26.9	2.1	*
OW2-SA4	-29.1	-2.1	*
SA2-OG2	-25.0	-2.0	*

Table S9: Listing of all significant pairwise comparisons of the logarithmized friction between the emulsions of the second generation at the speed of 200 µm s^{-1}. The results of all pairwise comparisons that are not listed are not significant. Statistical analyses: Kruskal-Wallis ANOVA followed by Kruskal-Wallis post hoc multiple comparisons.

compared emulsions	test statistics	standardized test statistics	statistical significance
OW2-OG2	-65.9	-4.9	***
OW2-squalane	-67.4	-5.1	***
OW2-SG2	82.1	6.0	***
glass-OG2	64.9	4.5	***
glass-squalane	66.4	4.6	***
glass-SG2	81.2	5.5	***
OA2-OG2	-51.4	-4.1	***
OA2-squalane	-52.9	-4.2	***
OA2-SG2	67.7	5.2	***
SA2-SG2	-61.9	-4.4	***
SW2-SG2	-53.0	-4.0	***
water-SG2	52.1	4.0	***
OW2-SG4	-44.0	-3.3	**
SA2-OG2	-45.6	-3.3	**
SA2-squalane	-47.1	-4.0	**
OW2-SA4	-41.7	-3.1	**
SA4-SG2	40.4	3.1	**
glass-SG4	43.0	3.0	**
SW2-squalane	-38.2	-3.0	**
SG4-SG2	38.2	3.0	**
water-squalane	-37.3	-2.9	**
SW2-OG2	-36.7	-2.8	**
glass-SA4	40.8	2.8	**
water-OG2	35.8	2.8	**
OA2-SG4	-29.5	-2.3	*
OW2-water	-30.0	-2.2	*
OW2-SW2	29.1	2.1	*
OA2-SA4	-27.2	-2.1	*
SA4-squalane	-25.7	-2.0	*
glass-water	29.1	2.0	*

Table S10: Listing of all significant pairwise comparisons of the logarithmized friction between the emulsions of the second generation at the speed of 500 µm s^{-1}. The results of all pairwise comparisons that are not listed are not significant. Statistical analyses: Kruskal-Wallis ANOVA followed by Kruskal-Wallis post hoc multiple comparisons.

compared emulsions	test statistics	standardized test statistics	statistical significance
OW2-SA2	47.5	3.5	***
OW2-OG2	-60.7	-4.6	***
OW2-squalane	-66.4	-5.2	***
OW2-SG2	81.4	6.2	***
glass-squalane	58.0	3.9	***
glass-SG2	73.0	4.9	***
OA2-OG2	-43.5	-3.5	***
OA2-squalane	-49.2	-4.1	***
OA2-SG2	64.2	5.2	***
SW2-squalane	-46.7	-3.5	***
SW2-SG2	-61.7	-4.5	***
water-SG2	51.5	3.9	***
glass-OG2	52.3	3.5	**
SG4-SG2	42.3	3.4	**
SA4-SG2	41.6	3.3	**
OW2-SG4	-39.1	-3.1	**
OW2-SA4	-39.8	-3.0	**
SW2-OG2	-41.1	-3.0	**
water-squalane	-36.5	-2.9	**
SA2-SG2	-33.9	-2.6	*
glass-SA2	39.1	2.5	*
OA2-SA2	30.3	2.4	*
water-OG2	30.8	2.4	*
SG4-squalane	-27.3	-2.3	*
OW2-water	-29.9	-2.2	*
SA4-squalane	-26.6	-2.1	*
glass-SA4	31.4	2.1	*
glass-SG4	30.7	2.1	*
SW2-SA2	-27.8	-2.0	*

Table S11: Listing of all pairwise comparisons of the logarithmized friction within the emulsions of the first generation relating to the three different velocities. Statistical analyses: Friedman test followed by by Friedman post hoc multiple comparisons. Abbreviations: n.s. = not significant.

compared emulsions	test statistics	standardized test statistics	statistical significance
emulsion VG50			
VG50_50-VG50_500	-0.7	-1.6	n.s.
VG50_50-VG50_200	-1.1	-2.5	*
VG50_500-VG50_200	0.4	0.9	n.s.
emulsion VP50			
VP50_50-VP50_200	-0.9	-2.0	*
VP50_50-VP50_500	-1.2	-2.7	*
VP50_200-VP50_500	-0.3	-0.7	n.s.
emulsion WG20			
WG20_500-WG20_200	1.1	2.4	*
WG20_500-WG20_50	1.9	4.0	***
WG20_200-WG20_50	0.8	1.7	n.s.
emulsion WP20			
WP20_50-WP20_200	-1.1	-2.5	*
WP20_50-WP20_500	-1.9	-4.3	***
WP20_200-WP20_500	-0.8	-1.8	n.s.
control water			
no significances between the three velocities			
control glass			
no significances between the three velocities			

Table S12: Listing of all pairwise comparisons of the logarithmized friction within the emulsions of the second generation and the squalane control relating to the three different velocities. Statistical analyses: Friedman test followed by Friedman post hoc multiple comparisons. Abbreviations: n.s. = not significant.

compared emulsions	test statistics	standardized test statistics	statistical significance
emulsion SW2			
SW2_50-SW2_500	-0.9	-1.6	n.s.
SW2_50-SW2_200	-1.7	-3.2	**
SW2_500-SW2_200	0.9	1.6	n.s.
emulsion OW2			
no significances between the three velocities			
emulsion SA2			
no significances between the three velocities			
emulsion SG2			
SG2_200-SG2_50	0.9	1.6	n.s.
SG2_200-SG2_500	-1.7	-3.2	**
SG2_50-SG2_500	-0.9	-1.6	n.s.
emulsion OA2			
no significances between the three velocities			
emulsion OG2			
OG2_500-OG2_200	0.4	0.9	n.s.
OG2_500-OG2_50	1.2	2.6	*
OG2_200-OG2_50	0.8	1.7	n.s.
emulsion SA4			
no significances between the three velocities			
emulsion SG4			
SG4_50-SG4_500	-1.0	-2.2	*
SG4_50-SG4_200	-1.7	-3.8	***
SG4_500-SG4_200	0.7	1.6	n.s.
control squalane			
squalane_50-squalane_200	-1.3	-2.9	**
squalane_50-squalane_500	-1.4	-3.1	**
squalane_200-squalane_500	-0.1	-0.2	n.s.
control water			
no significances between the three velocities			
control glass			
no significances between the three velocities			

Table S13: Listing of all significant correlations (Pearson) between logarithmized structural and chemical parameters.

		phase volume ratio	10% quantile	25% quantile	75% quantile	90% quantile	arithmetic mean	median	mean/median	modus	SD	excess kurtosis
Vaseline	statistical significance			*	*	*	*	*		**	*	
	correlation			0.60	0.58	0.61	0.62	0.61		0.68	0.65	
squalane	statistical significance	(*) (0.09)										
	correlation	0.47										
octacosane	statistical significance											*
	correlation											0.56
glycerine	statistical significance				*	*	*	*			**	
	correlation				0.62	0.64	0.61	0.57			0.72	
SDS	statistical significance			*	**	**	**	**		**	**	
	correlation			0.59	0.73	0.76	0.75	0.76		0.69	0.80	
Span 80	statistical significance	**	**									
	correlation	0.68	0.69									
AOT	statistical significance	**	*									
	correlation	0.82	0.58									
water	statistical significance								(*) (0.07)			
	correlation								0.50			

Table S14: Listing of all significant correlations (Pearson) between logarithmized structural and tribological parameters.

		phase volume ratio	25% quantile	75% quantile	90% quantile	arithmetic mean	median	modus	SD	excess kurtosis
adhesion	statistical significance	(*) (0.01)		*	*	*	**		*	(*) (0.07)
	correlation	0.46		0.61	0.63	0.63	0.71		0.56	-0.50
friction 50µm/s	statistical significance			**	**	**	**		**	*
	correlation			0.73	0.74	0.73	0.74		0.77	-0.56
friction 200µm/s	statistical significance			**	**	**	**	(*) (0.05)	**	*
	correlation			0.77	0.78	0.77	0.78	0.53	0.82	-0.59
friction 500µm/s	statistical significance		(*) (0.08)	**	**	**	**	*	**	*
	correlation		0.48	0.74	0.75	0.75	0.77	0.56	0.77	-0.58

Table S15: Listing of all significant correlations (Pearson) between logarithmized tribological and chemical parameters.

		Vaseline	microcrystalline wax	poly(vinyl alcohol)	gelatin	glycerine	SDS	Span 80
adhesion	statistical significance	(*) (0.06)	(*) (0.06)	*	(*) (0.09)		**	
	Correlation	0.51	0.51	0.59	0.47		0.68	
friction 50µm/s	statistical significance	(*) (0.06)	*		**	**	**	(*) (0.07)
	correlation	0.52	0.57		0.72	0.85	0.73	-0.50
friction 200µm/s	statistical significance	*	*		(*) (0.06)	**	**	*
	correlation	0.65	0.57		0.52	0.75	0.86	-0.54
friction 500µm/s	statistical significance	*	*	*	(*) (0.10)	*	**	*
	correlation	0.65	0.56	0.57	0.46	0.62	0.85	-0.54

Table S16: Densities of used compounds for calculation of the volumes of both hydrophilic and hydrophobic phases.

**compound**	**density [g/ml]**
glycerin	1.249 (25°C) Sigma Aldrich
squalane	0.81
sorbitanmonooleat (Span 80)	0.99 (molecular mass: 428.26 g/mol; molecular mass of oleat = 281.46 g/mol)
Vaseline (petrolatum)	1
microcrystalline wax	1
ocatacosane	1
albumin	1
gelatin	1
H_2O	1
